# Supplementary material for: Cloud drop number concentrations over the western North Atlantic Ocean: seasonal cycle, aerosol interrelationships, and other influential factors
Source: Atmos Chem Phys. Author manuscript; Available in PMC 2021 Aug 9. (PMC8350960; doi:10.5194/acp-21-10499-2021)
Supplement: Supplement [file NIHMS1724114-supplement-Supplement.pdf]

Supplement of Atmos. Chem. Phys., 21, 10499–10526, 2021
https://doi.org/10.5194/acp-21-10499-2021-supplement
© Author(s) 2021. CC BY 4.0 License.

Atmospheric Chemistry and Physics
Open Access

*Supplement of*

# Cloud drop number concentrations over the western North Atlantic Ocean: seasonal cycle, aerosol interrelationships, and other influential factors

**Hossein Dadashazar et al.**

*Correspondence to:* Hossein Dadashazar (hosseind@arizona.edu)

The copyright of individual parts of the supplement might differ from the article licence.

**Table S1. Range of model hyperparameters tested during training/validation of the GBRT models through a combination of grid and random searches. Final model values are also listed in the last column.**

| Model parameter | Range of values tested | Final model values (DJF/JJA) |
|---|---|---|
| Learning rate | 0.001-0.1 | 0.05/0.05 |
| Number of estimators | 100-5000 | 400/400 |
| Maximum depth of a tree | 2-35 | 9/11 |
| Minimum number of samples to split an internal node | 20-100 | 66/45 |
| Minimum number of samples at a leaf node | 20-60 | 31/66 |

**Table S2: Mean seasonal values of speciated AOD and surface mass concentration for black carbon, dust, organic carbon, sulfate, and sea-salt for the six sub-domains in Figure S1.**

| Speciated AOD/Surface mass concentration (μg m$^{-3}$) | | | | | | |
|---|---|---|---|---|---|---|
| | S | C-S | C | C-N | N | Bermuda |
| Sulfate | | | | | | |
| DJF | 0.03/0.91 | 0.05/1.37 | 0.05/1.49 | 0.06/1.99 | 0.06/1.50 | 0.03/0.59 |
| MAM | 0.05/0.99 | 0.06/1.37 | 0.07/1.54 | 0.08/2.38 | 0.07/1.84 | 0.04/0.72 |
| JJA | 0.05/0.75 | 0.07/1.10 | 0.07/1.25 | 0.09/2.26 | 0.08/1.45 | 0.04/0.50 |
| SON | 0.04/0.73 | 0.04/1.00 | 0.04/1.08 | 0.05/1.56 | 0.05/1.09 | 0.02/0.41 |
| Sea-salt | | | | | | |
| DJF | 0.04/38.10 | 0.04/39.12 | 0.05/45.89 | 0.03/29.55 | 0.03/27.45 | 0.05/49.51 |
| MAM | 0.04/36.72 | 0.04/38.04 | 0.04/44.28 | 0.03/28.52 | 0.02/23.22 | 0.04/41.76 |
| JJA | 0.05/46.12 | 0.05/48.83 | 0.05/56.56 | 0.03/30.91 | 0.02/17.99 | 0.03/35.95 |
| SON | 0.06/48.44 | 0.05/43.76 | 0.06/54.24 | 0.04/36.20 | 0.03/27.24 | 0.06/54.60 |
| Dust | | | | | | |
| DJF | <0.01/0.74 | <0.01/1.06 | 0.01/1.64 | 0.01/2.64 | 0.01/1.24 | <0.01/0.98 |
| MAM | 0.02/3.90 | 0.02/3.86 | 0.02/4.03 | 0.03/4.47 | 0.02/2.90 | 0.02/2.81 |
| JJA | 0.03/12.05 | 0.02/8.22 | 0.02/6.08 | 0.02/5.02 | 0.02/2.41 | 0.02/6.58 |
| SON | 0.01/2.56 | 0.01/1.79 | 0.01/2.01 | 0.01/2.51 | 0.01/1.20 | 0.01/2.01 |
| Organic carbon | | | | | | |
| DJF | 0.01/0.69 | 0.02/0.92 | 0.02/0.67 | 0.02/1.07 | 0.02/0.58 | 0.01/0.25 |
| MAM | 0.03/1.00 | 0.04/1.37 | 0.04/1.02 | 0.04/1.53 | 0.03/0.92 | 0.03/0.42 |
| JJA | 0.01/0.55 | 0.03/1.16 | 0.03/1.25 | 0.05/2.49 | 0.05/2.43 | 0.02/0.35 |
| SON | 0.01/0.67 | 0.02/1.03 | 0.02/0.87 | 0.02/1.43 | 0.02/0.98 | 0.01/0.34 |
| Black carbon | | | | | | |
| DJF | <0.01/0.10 | <0.01/0.15 | 0.01/0.15 | 0.01/0.37 | 0.01/0.20 | <0.01/0.08 |
| MAM | 0.01/0.12 | 0.01/0.16 | 0.01/0.17 | 0.01/0.38 | 0.01/0.21 | 0.01/0.09 |
| JJA | <0.01/0.08 | 0.01/0.12 | 0.01/0.15 | 0.01/0.41 | 0.01/0.31 | <0.01/0.06 |
| SON | <0.01/0.09 | <0.01/0.12 | <0.01/0.14 | 0.01/0.32 | 0.01/0.20 | <0.01/0.06 |

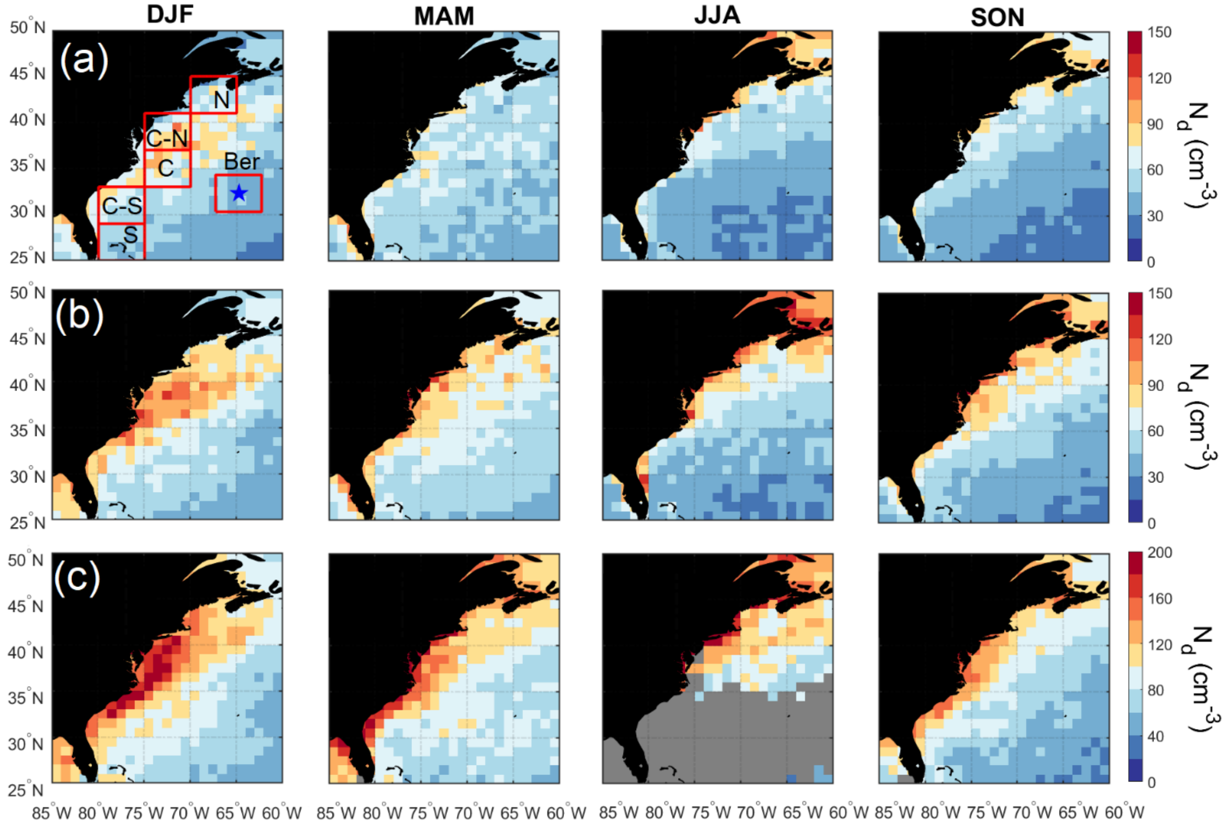


**Figure S1: Seasonal maps of cloud drop number concentration for different ranges of low-level liquid cloud fraction ($CF_{low\text{-}liq.}$) as follows: (a) $0.1 \leq CF_{low\text{-}liq.} < 0.3$, (b) $0.3 \leq CF_{low\text{-}liq.} < 0.6$, and (c) $CF_{low\text{-}liq.} \geq 0.7$. Gray pixels represent regions without sufficent sample points (less than 10 points) for calculating averages.**

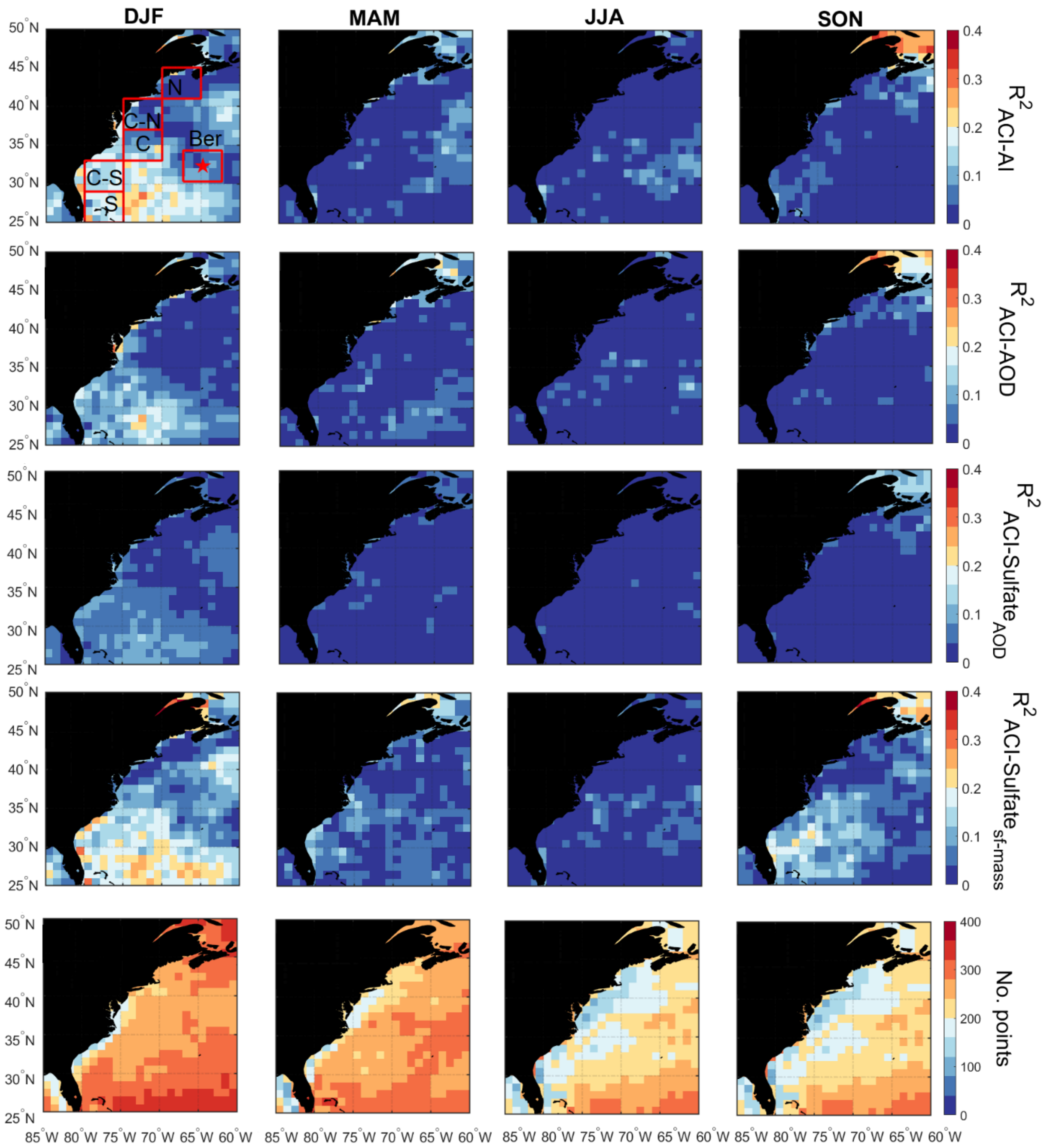


**Figure S2: Seasonal maps of the coefficient of determination ($R^2$) and number of points for the analysis of the aerosol-cloud interaction (ACI) parameters over the WNAO using daily $N_d$ and four different aerosol proxy parameter values (AI, AOD, Sulfate$_{AOD}$, Sulfate$_{sf\text{-}mass}$) from CERES-MODIS and MERRA-2, respectively. ACI statistics associated with the six sub-domains shown are summarized in Table 4.**

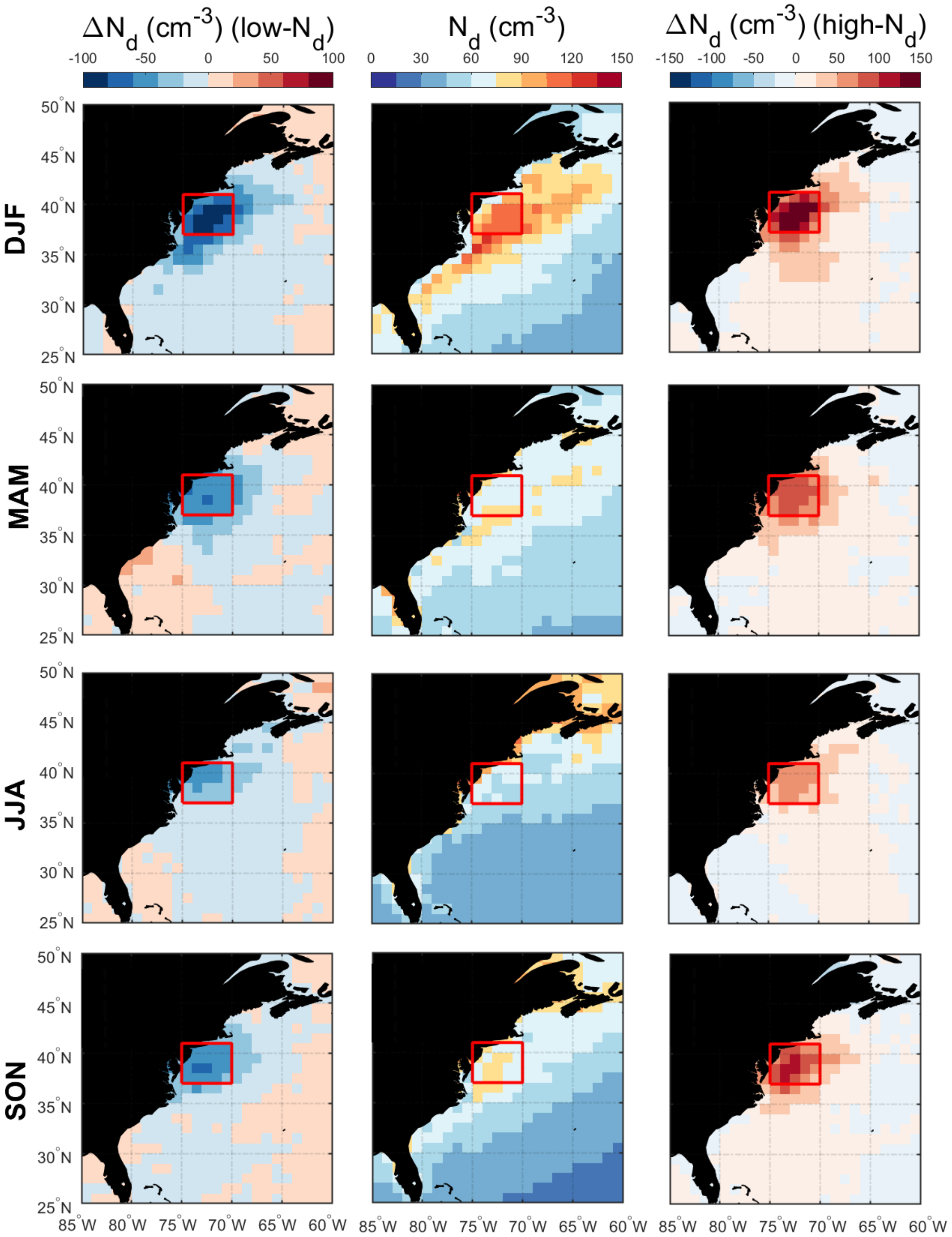


**Figure S3: Seasonal climatology of $N_d$ (middle column) and anomalies from seasonal averages for low-$N_d$ days (left column) and high-$N_d$ days (right column). The red box represents sub-domain C-N for which the analysis was conducted, as explained in Section 4.1.**

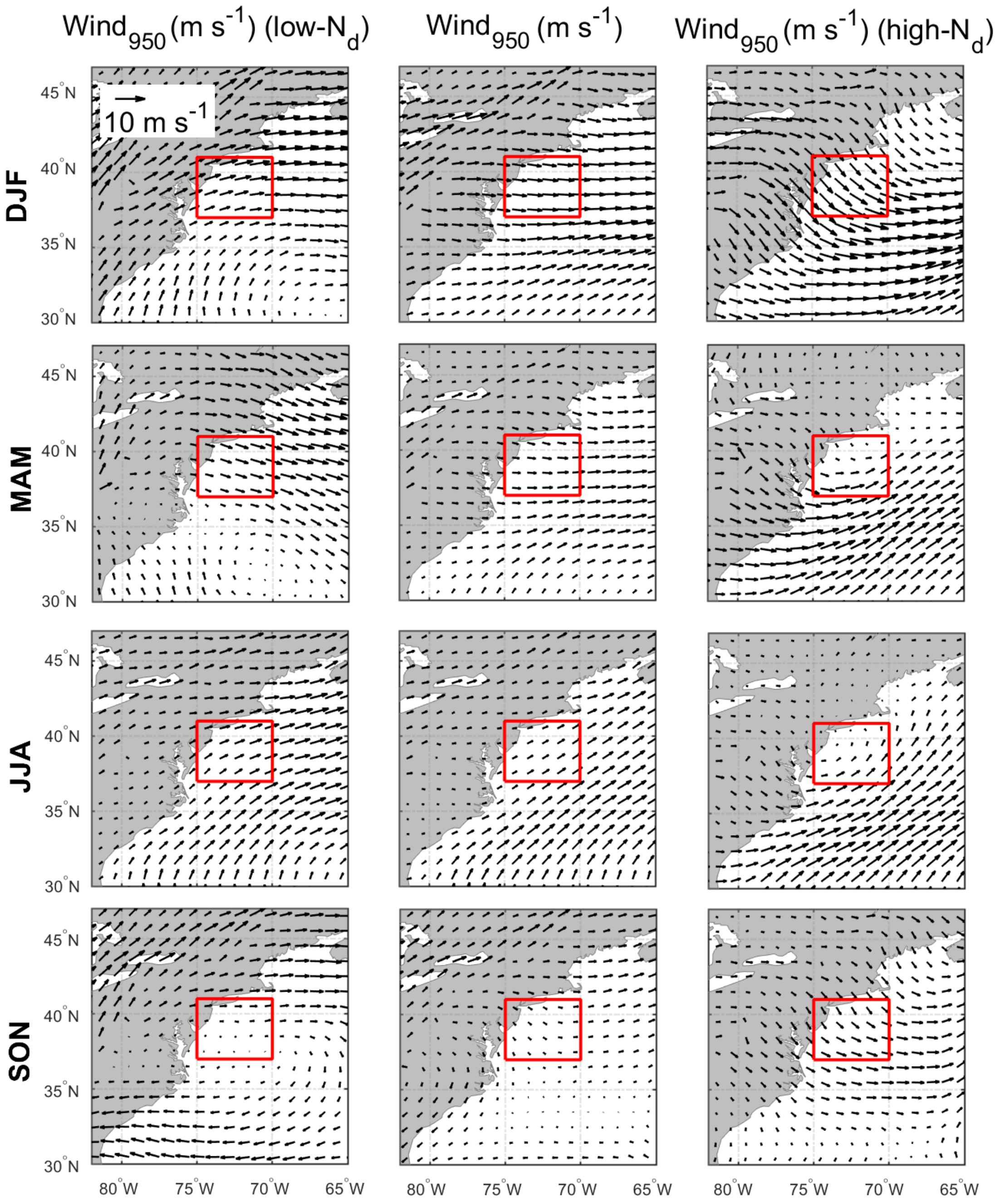


**Figure S4: Seasonal climatology of winds at 950 hPa (middle column) and anomalies from seasonal averages for low-N$_d$ days (left column) and high-N$_d$ days (right column). The reference wind vector is shown on the top left panel. The red box represents sub-domain C-N for which the analysis was conducted.**

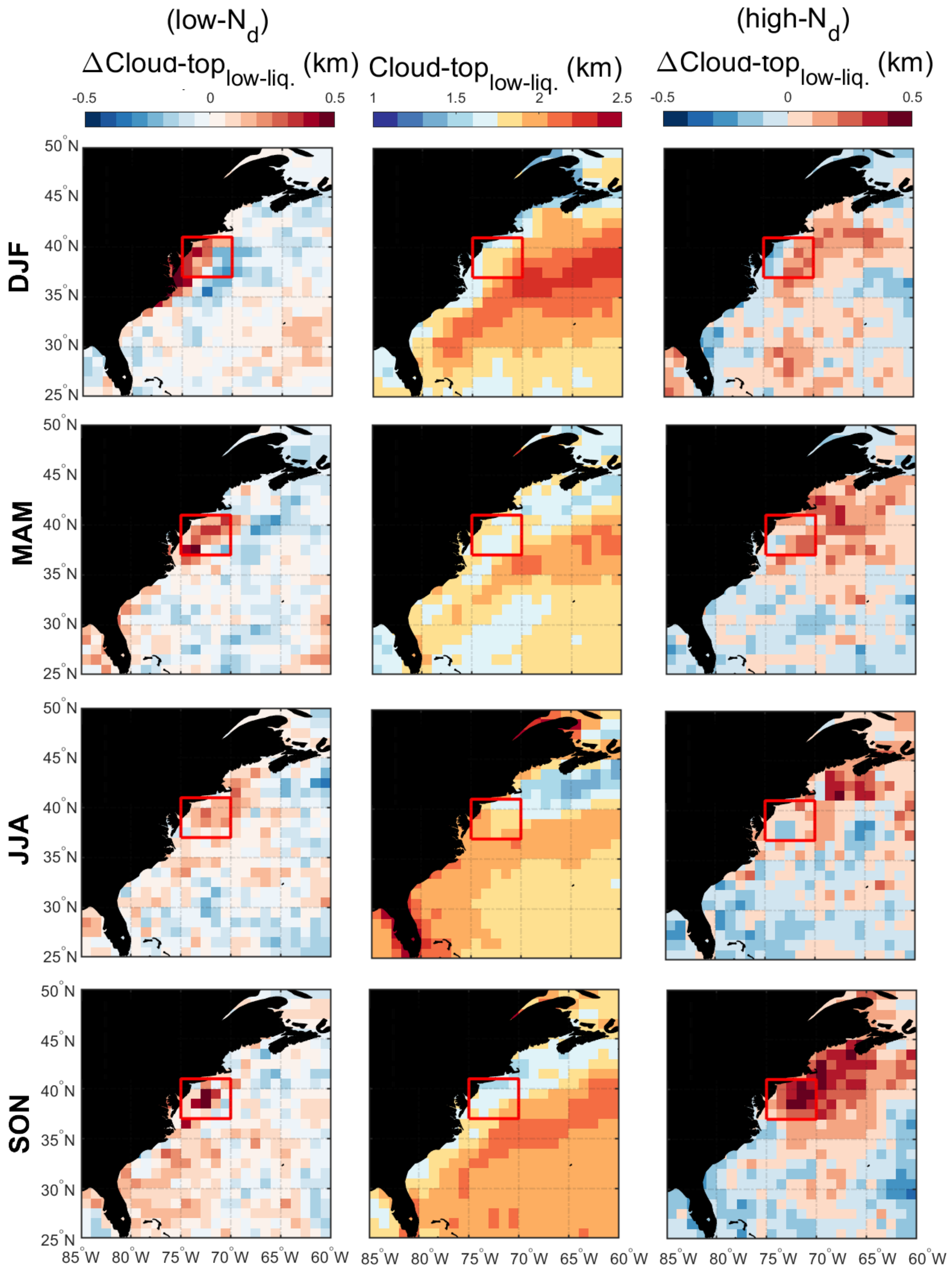


**Figure S5: Seasonal averages of low-level liquid cloud-top effective height (middle column) and associated anomalies on low-$N_d$ days (left column) and high-$N_d$ days (right column). The red box represents sub-domain C-N for which the analysis was conducted.**

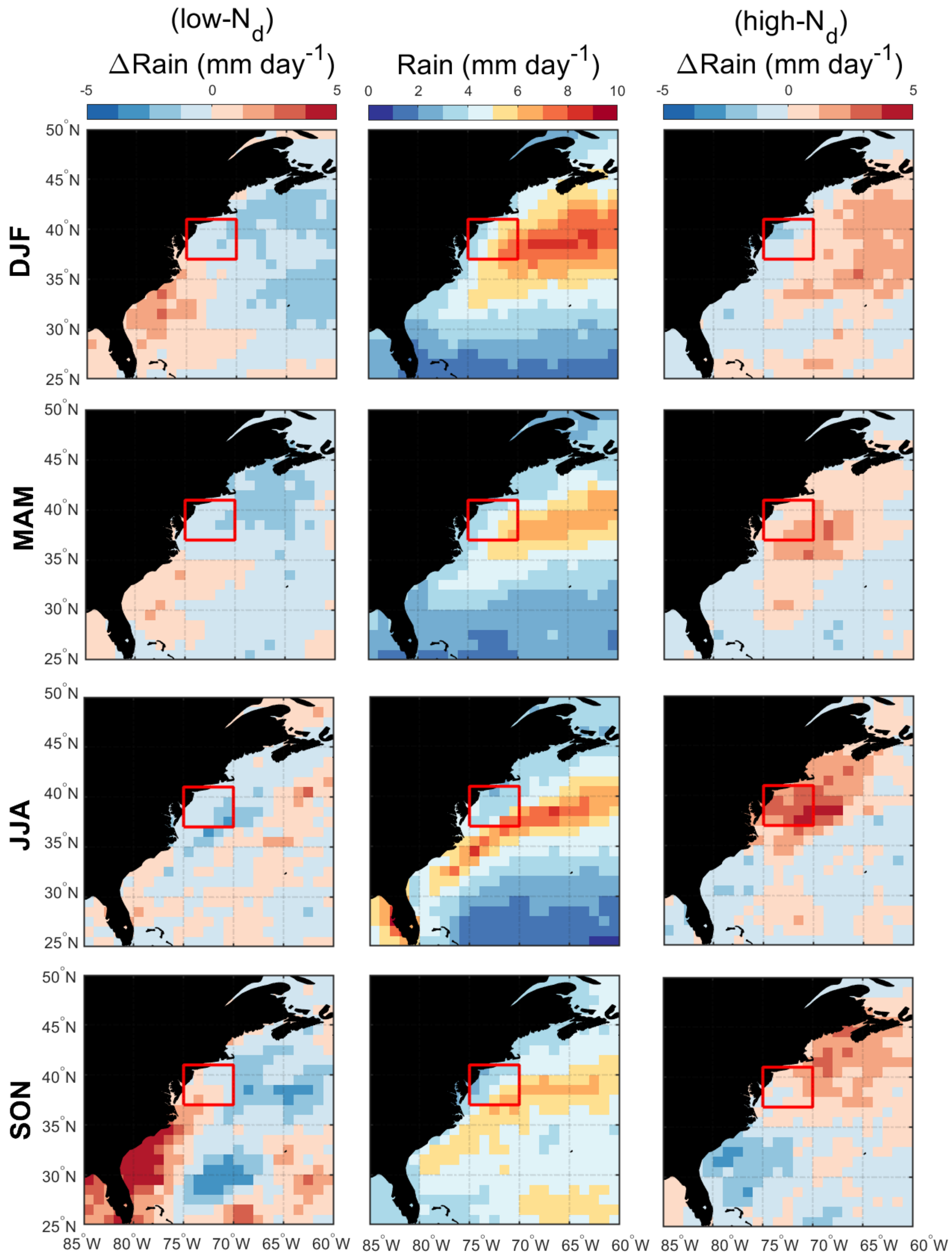


68
69 **Figure S6: Seasonal averages of PERSIANN-CDR precipitation rate (middle column) and**
70 **associated anomalies on low-$N_d$ days (left column) and high-$N_d$ days (right column). The**
71 **red box represents sub-domain C-N for which the analysis was conducted.**
72

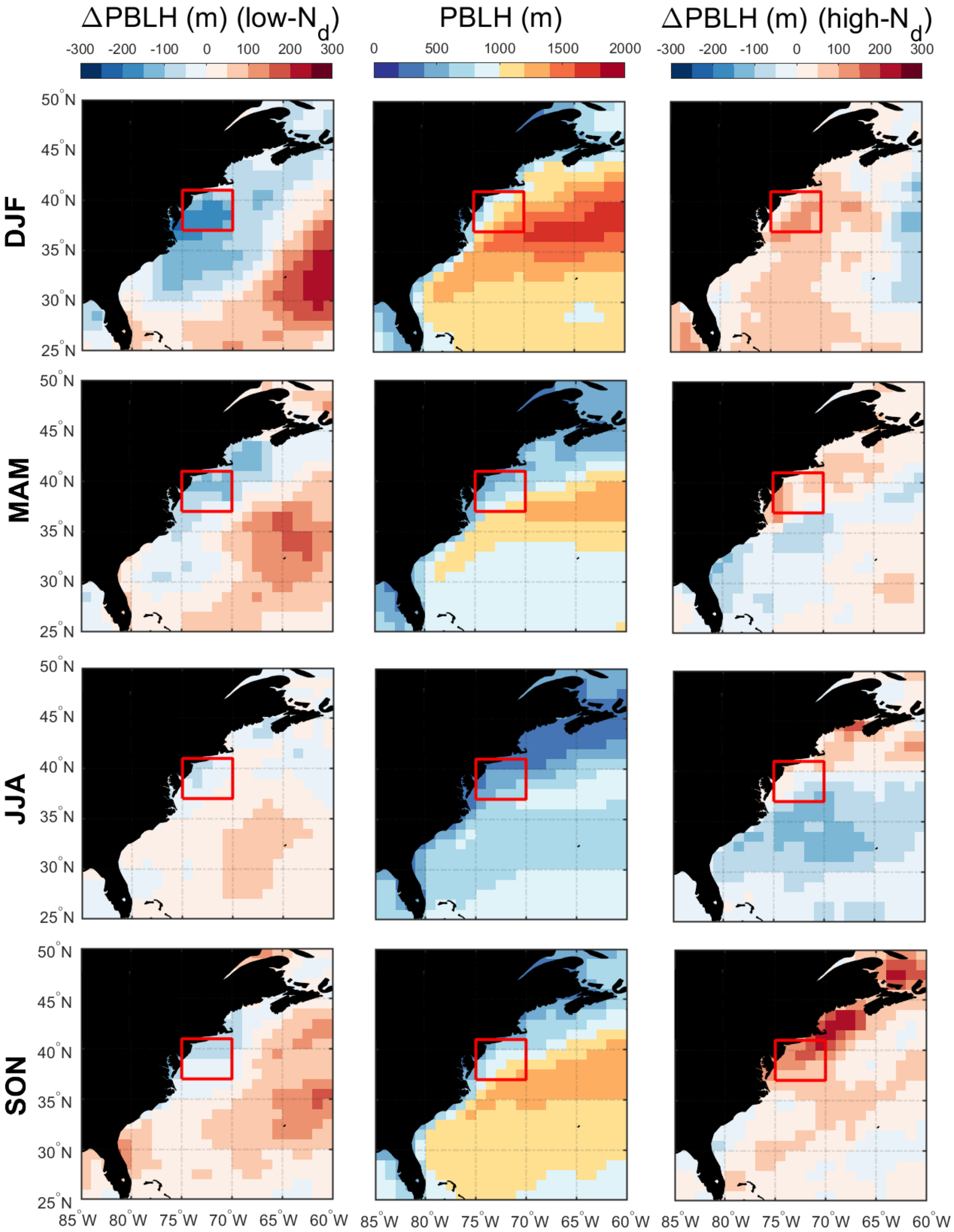


**Figure S7: Seasonal averages of planetary boundary layer height (middle column) and associated anomalies on low-$N_d$ days (left column) and high-$N_d$ days (right column). The red box represents sub-domain C-N for which the analysis was conducted.**

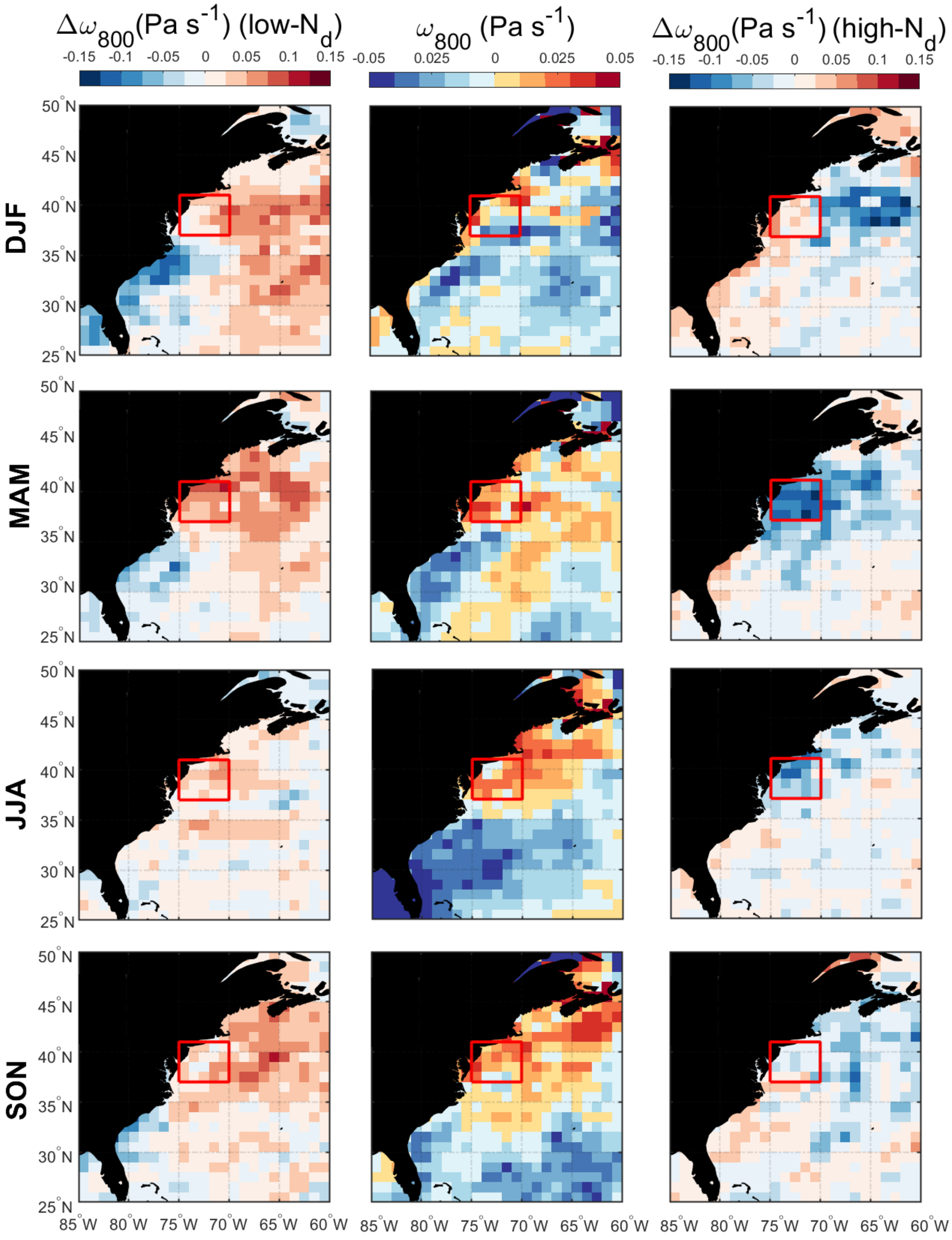


**Figure S8: Seasonal averages of vertical pressure velocity at 800 hPa (middle column) and associated anomalies on low-N$_d$ days (left column) and high-N$_d$ days (right column). The red box represents sub-domain C-N for which the analysis was conducted.**

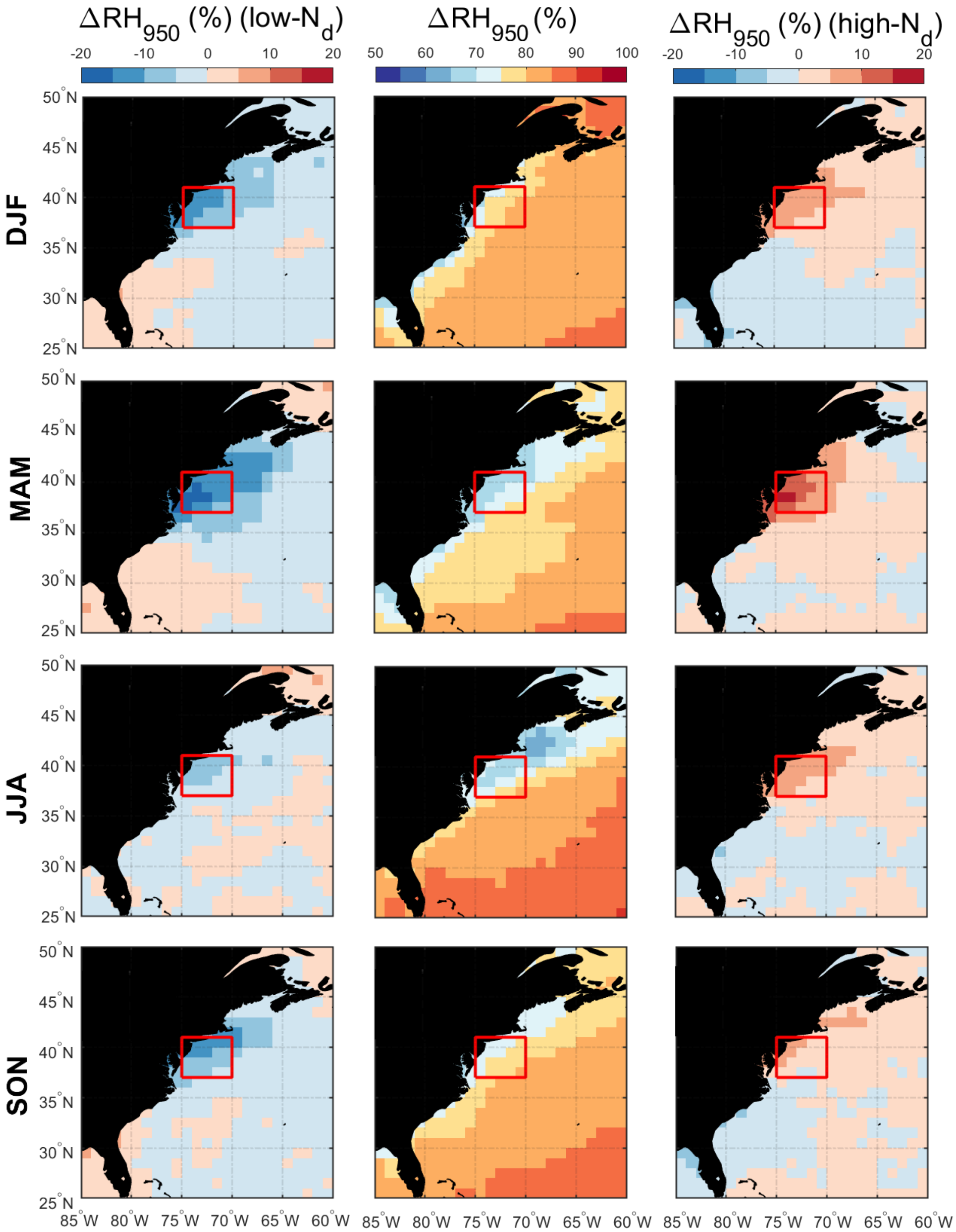


**Figure S9: Seasonal averages of relative humidity at 950 hPa (middle column) and associated anomalies on low-$N_d$ days (left column) and high-$N_d$ days (right column). The red box represents sub-domain C-N for which the analysis was conducted.**

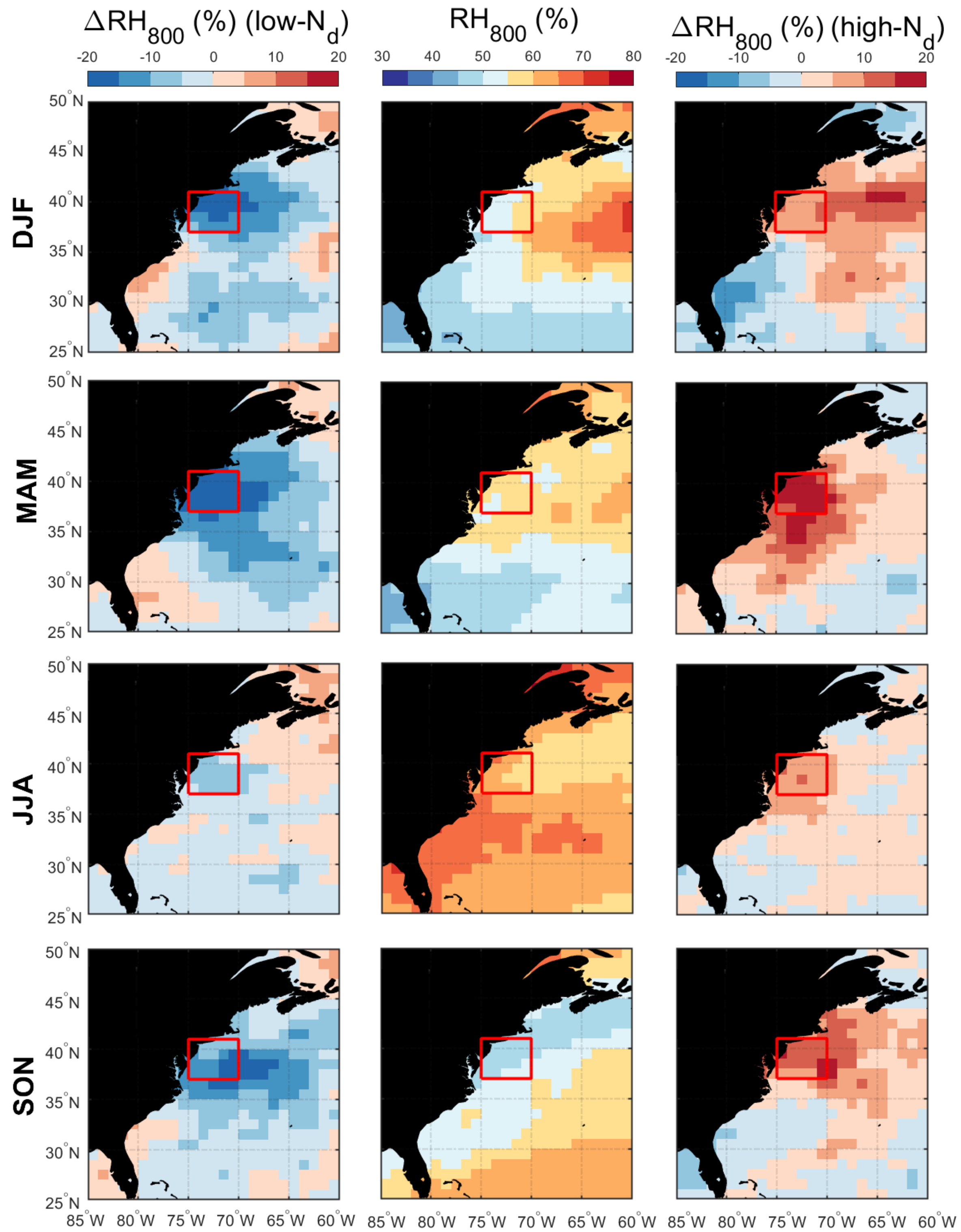


**Figure S10: Seasonal averages of relative humidity at 800 hPa (middle column) and associated anomalies on low-$N_d$ days (left column) and high-$N_d$ days (right column). The red box represents sub-domain C-N for which the analysis was conducted.**

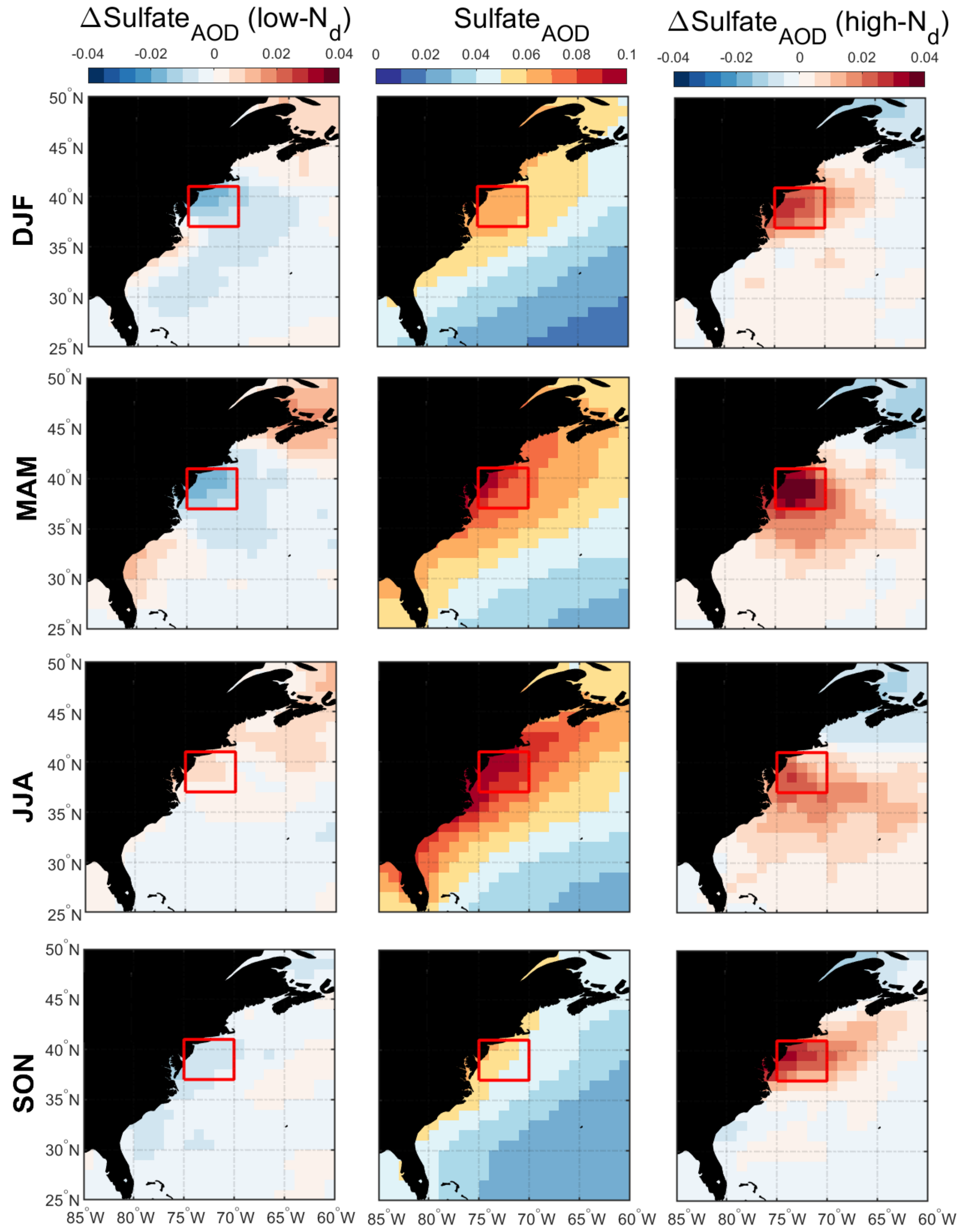


**Figure S11: Seasonal averages of sulfate AOD (middle column) and associated anomalies on low-$N_d$ days (left column) and high-$N_d$ days (right column). The red box represents sub-domain C-N for which the analysis was conducted.**

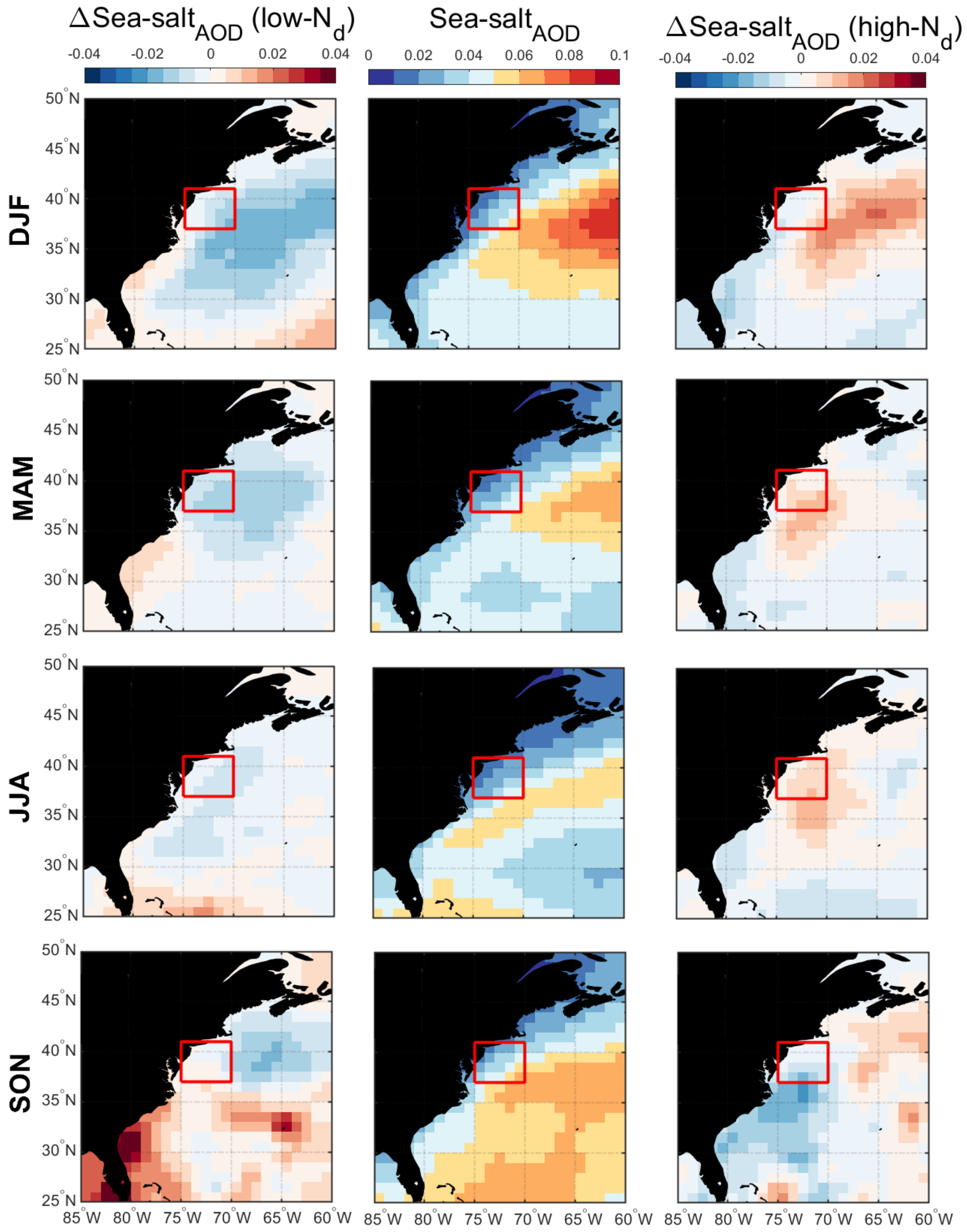


**Figure S12: Seasonal averages of sea-salt AOD (middle column) and associated anomalies on low-$N_d$ days (left column) and high-$N_d$ days (right column). The red box represents sub-domain C-N for which the analysis was conducted.**

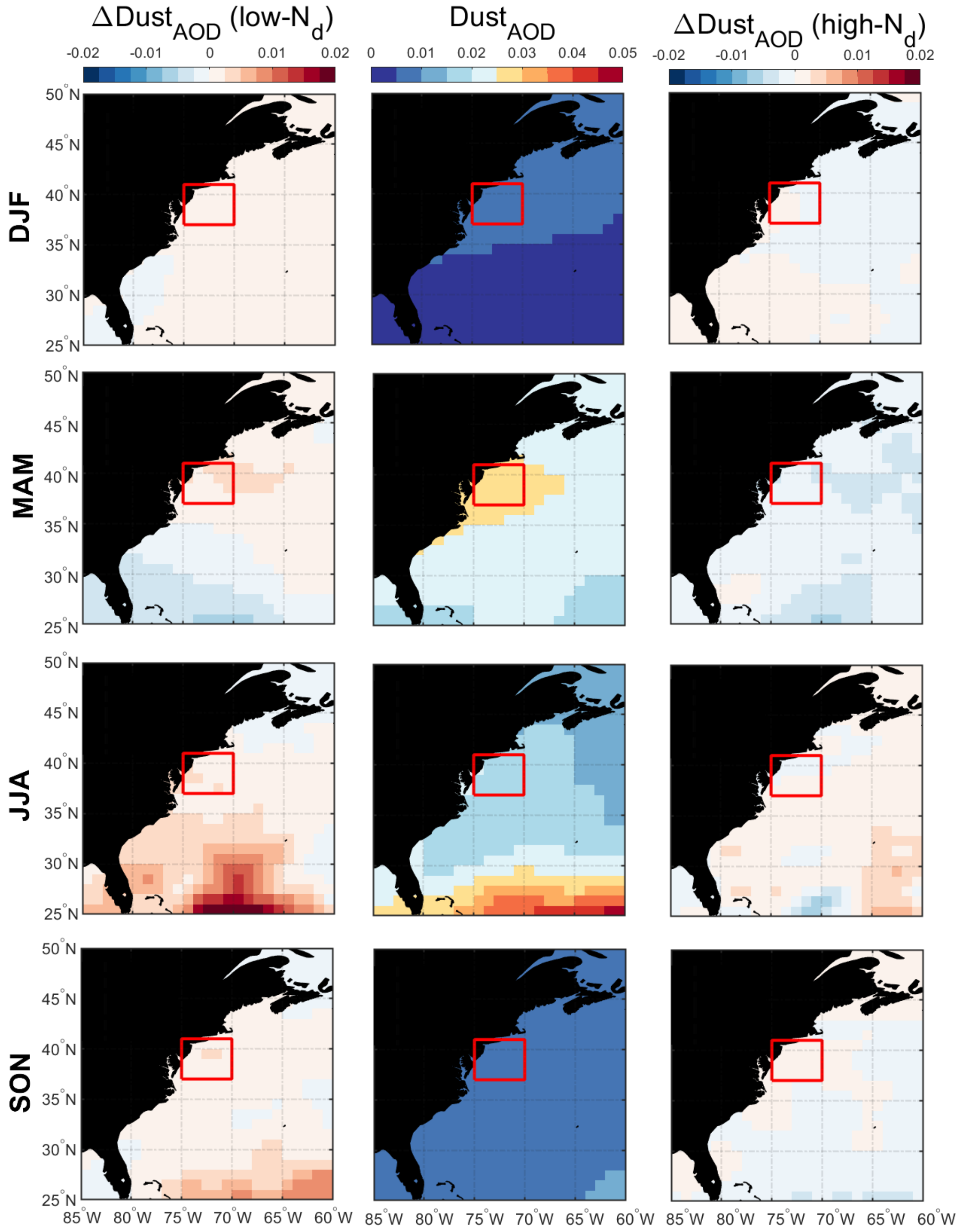


**Figure S13: Seasonal averages of dust AOD (middle column) and associated anomalies on low-$N_d$ days (left column) and high-$N_d$ days (right column). The red box represents sub-domain C-N for which the analysis was conducted.**

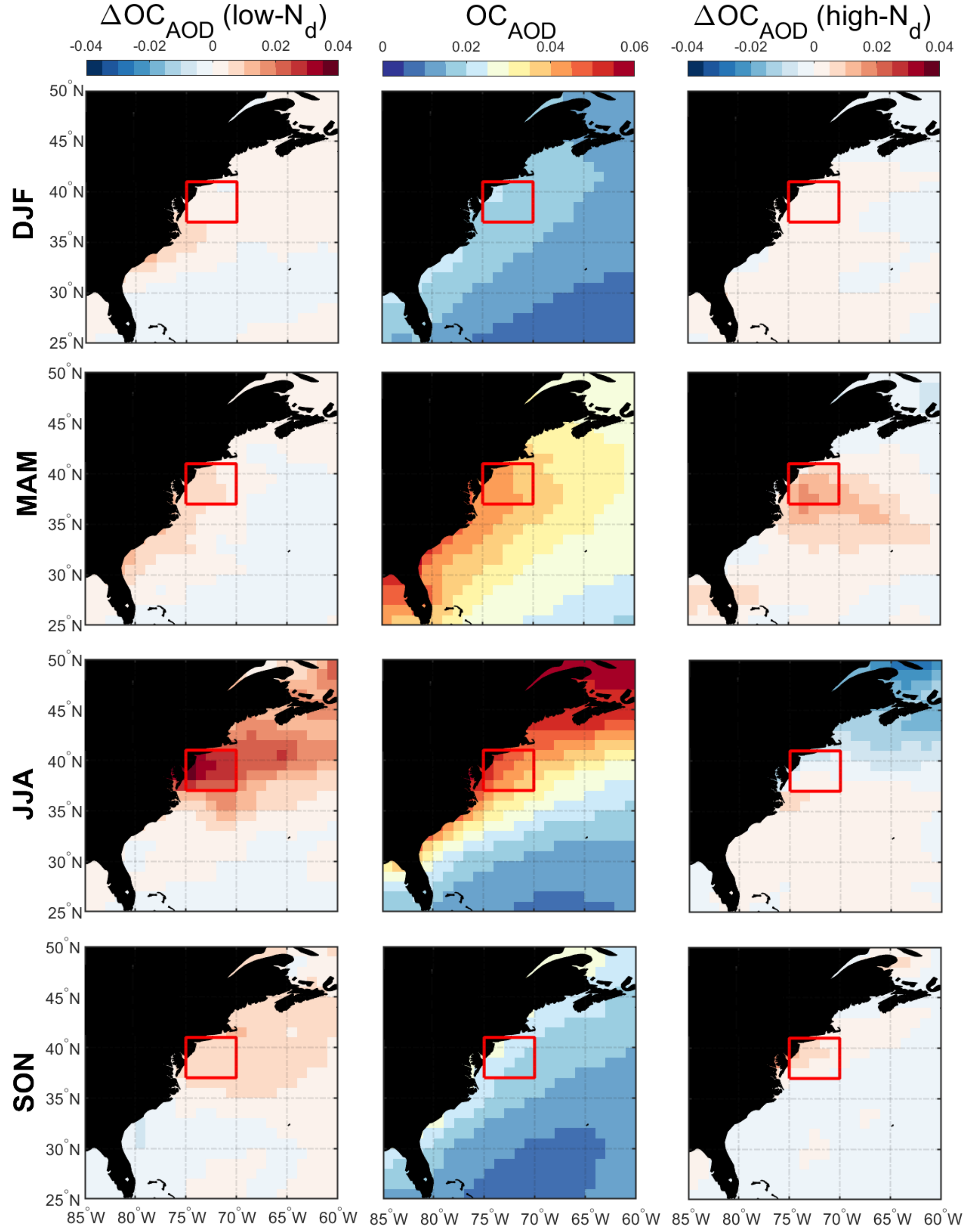


**Figure S14: Seasonal averages of organic carbon AOD (middle column) and associated anomalies on low-$N_d$ days (left column) and high-$N_d$ days (right column). The red box represents sub-domain C-N for which the analysis was conducted.**

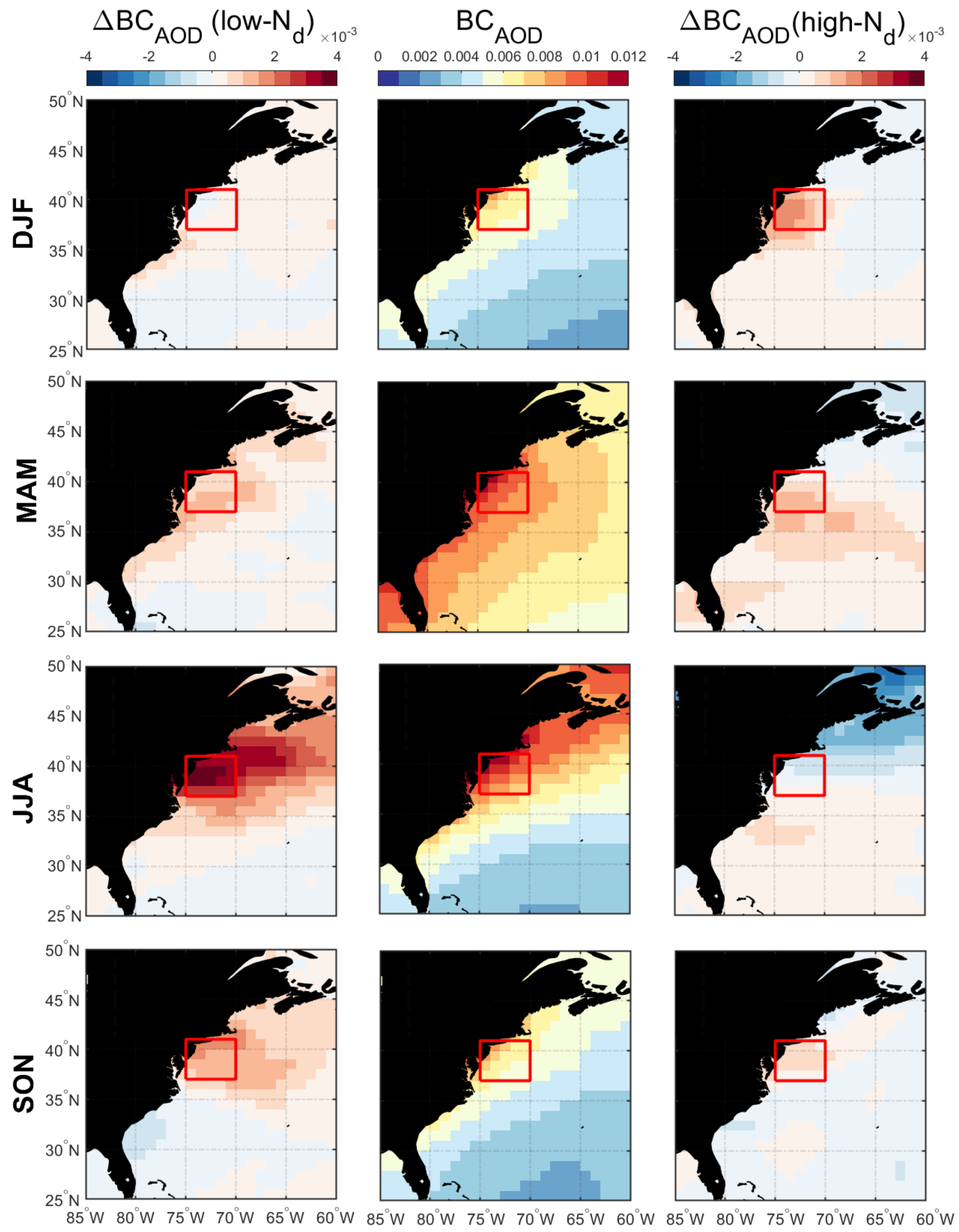


**Figure S15: Seasonal averages of black carbon AOD (middle column) and associated anomalies on low-$N_d$ days (left column) and high-$N_d$ days (right column). The red box represents sub-domain C-N for which the analysis was conducted.**

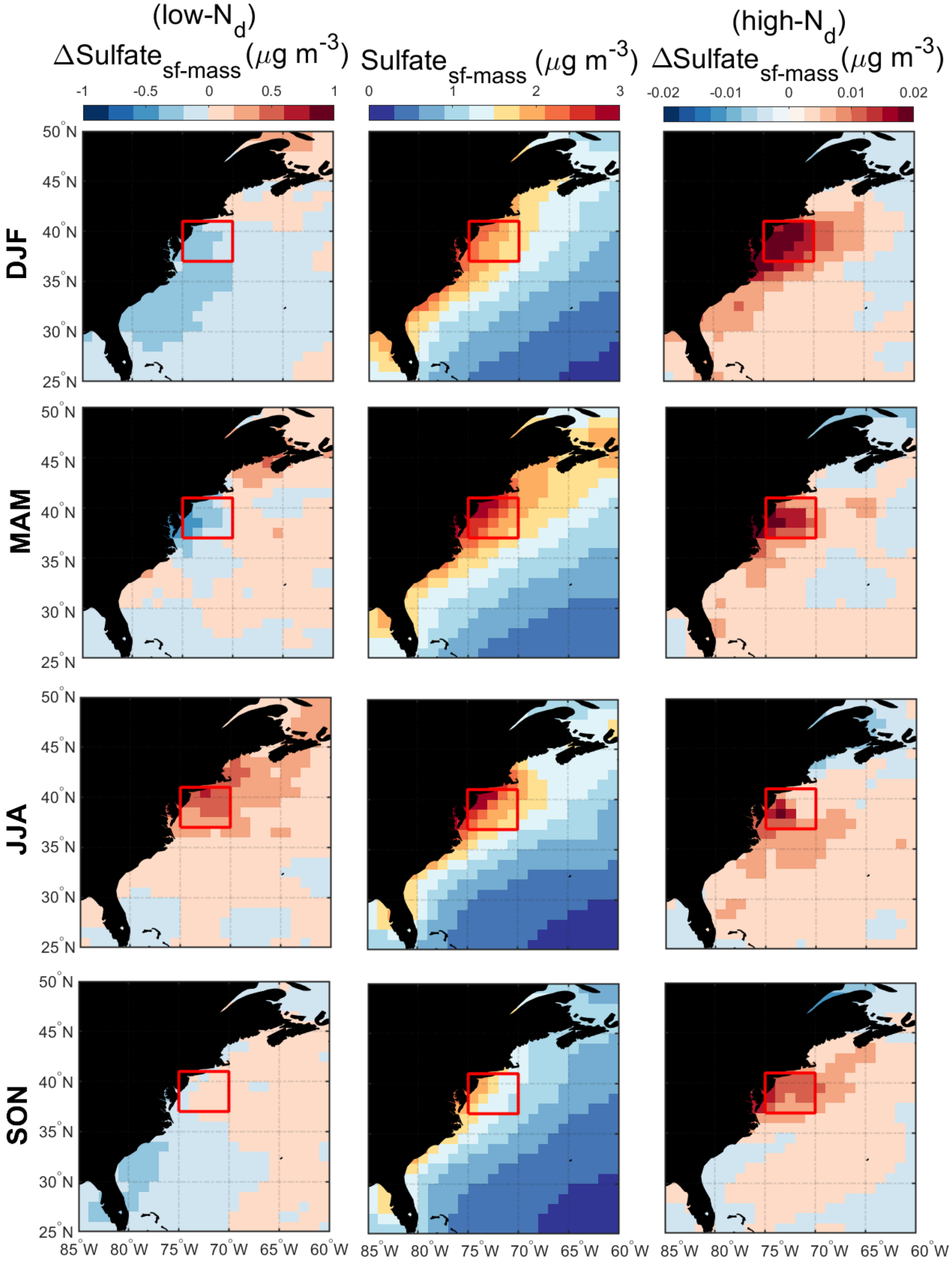


**Figure S16: Seasonal averages of sulfate surface mass concentration (middle column) and associated anomalies on low-$N_d$ days (left column) and high-$N_d$ days (right column). The red box represents sub-domain C-N for which the analysis was conducted.**

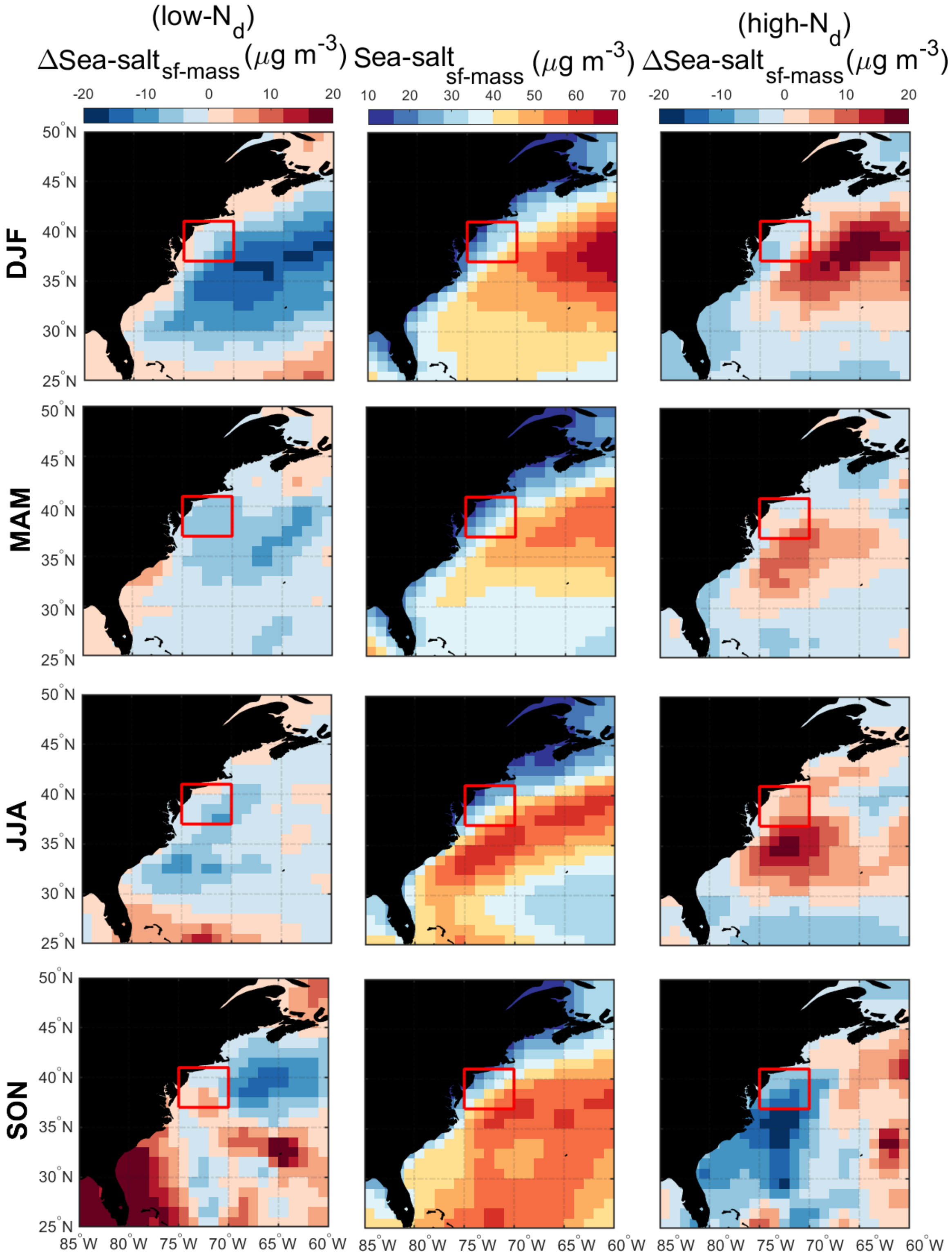


**Figure S17: Seasonal averages of sea-salt surface mass concentration (middle column) and associated anomalies on low-N$_d$ days (left column) and high-N$_d$ days (right column). The red box represents sub-domain C-N for which the analysis was conducted.**

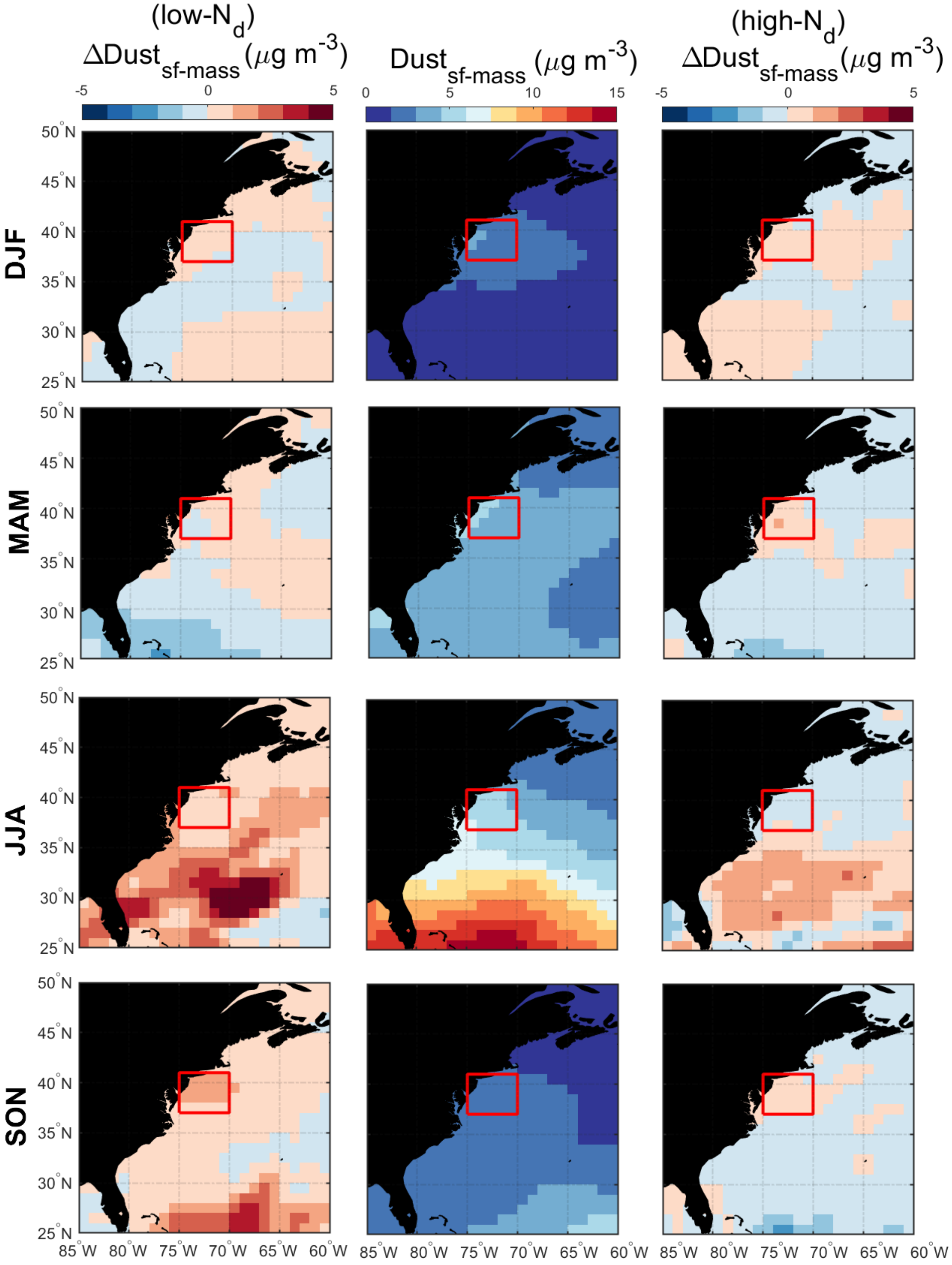


**Figure S18: Seasonal averages of dust surface mass concentration (middle column) and associated anomalies on low-N$_d$ days (left column) and high-N$_d$ days (right column). The red box represents sub-domain C-N for which the analysis was conducted.**

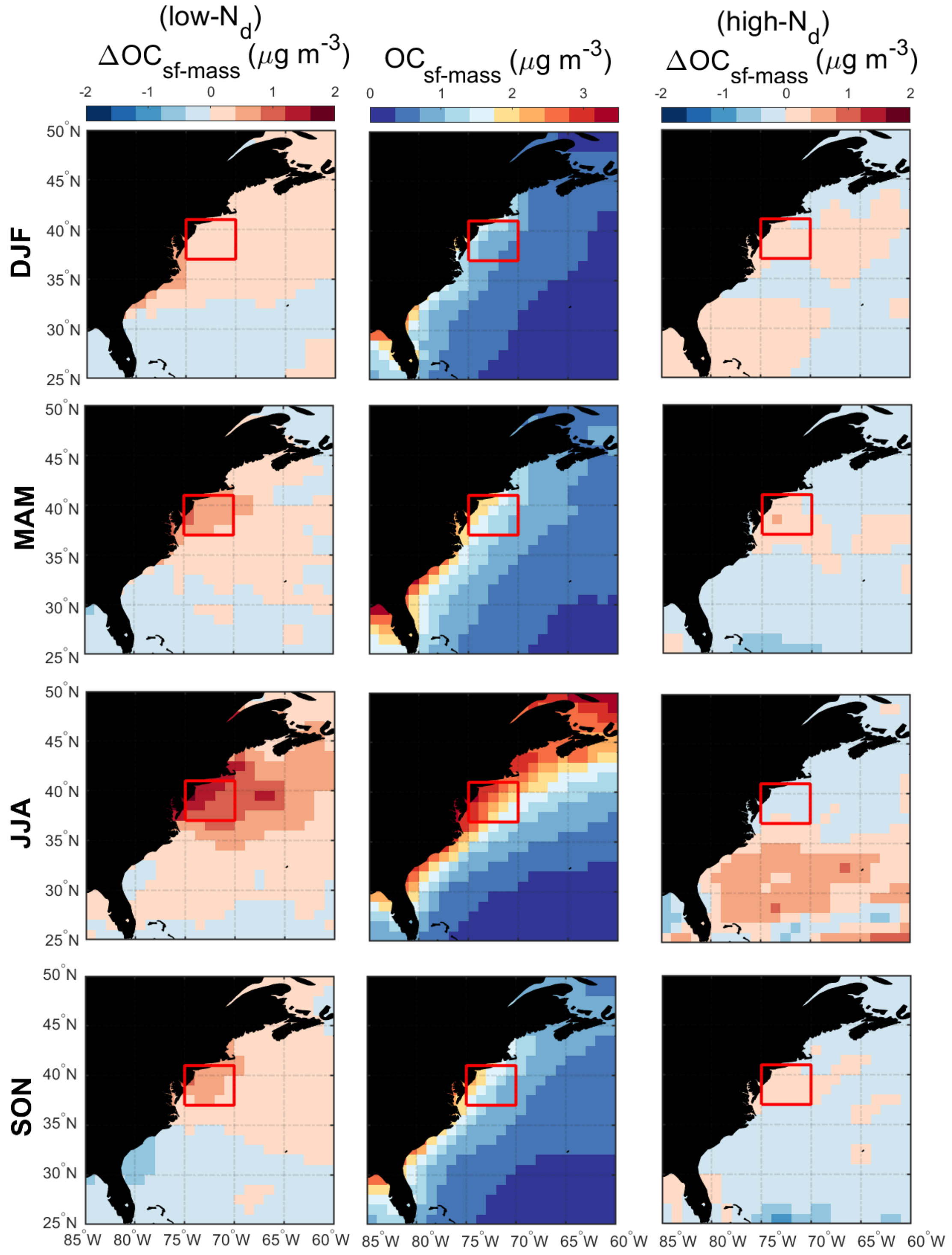


**Figure S19: Seasonal averages of organic carbon (OC) surface mass concentration (middle column) and associated anomalies on low-$N_d$ days (left column) and high-$N_d$ days (right column). The red box represents sub-domain C-N for which the analysis was conducted.**

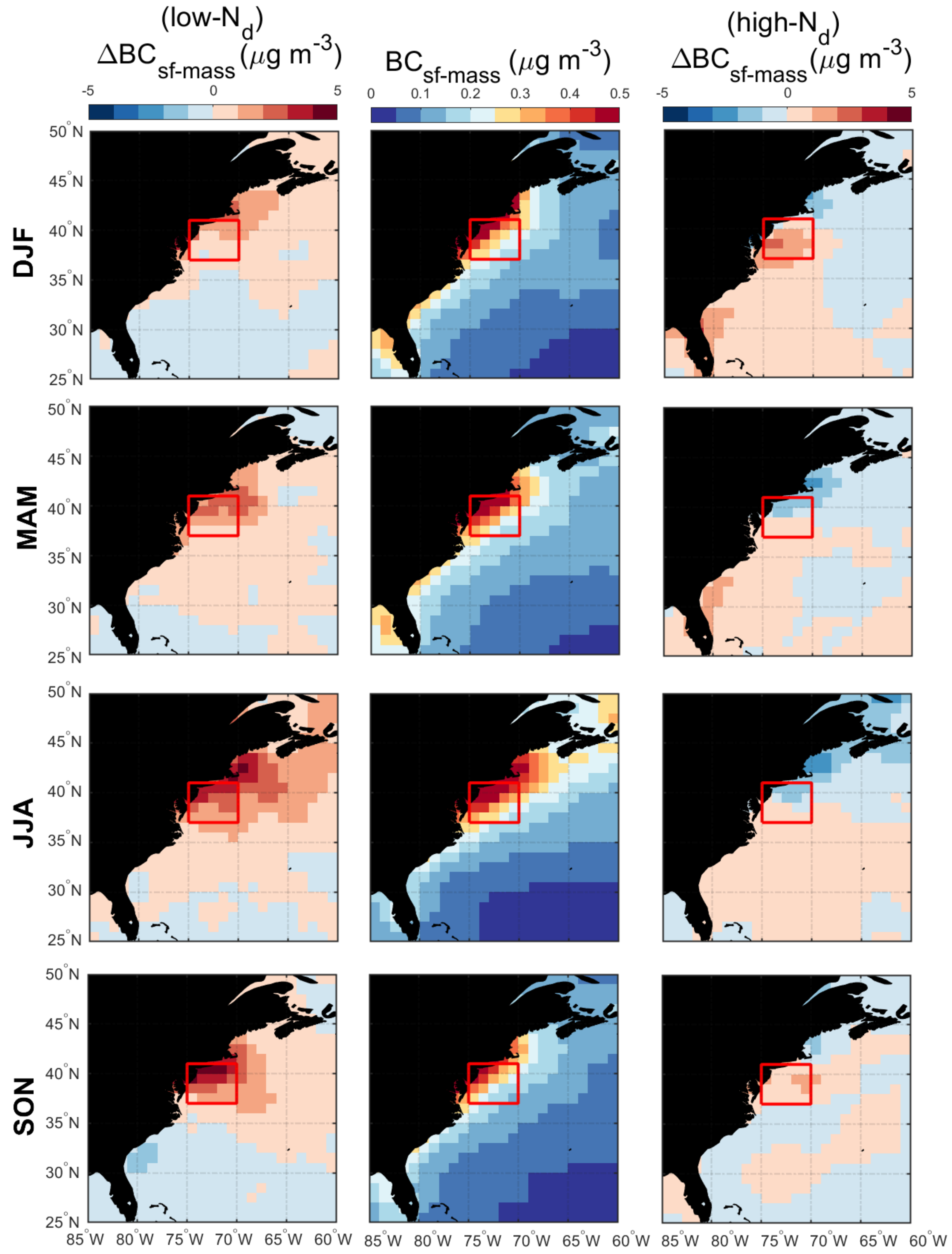


**Figure S20: Seasonal averages of black carbon (BC) surface mass concentration (middle column) and associated anomalies on low-$N_d$ days (left column) and high-$N_d$ days (right column). The red box represents sub-domain C-N for which the analysis was conducted.**

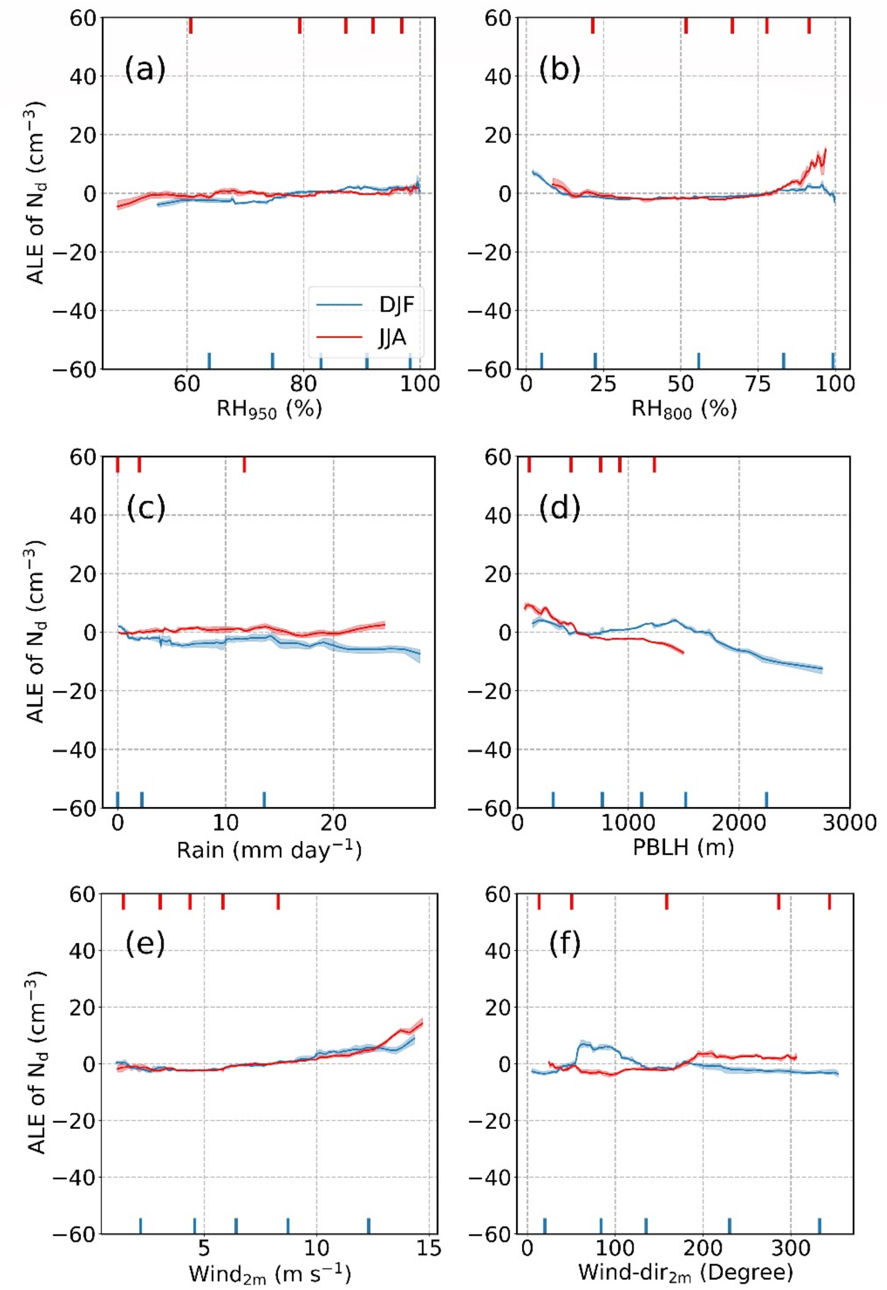


**Figure S21: Average local accumulated effect (ALE) profiles based on GBRT modeling of the following parameters: (a) relative humidity at 950 hPa ($RH_{950}$), (b) relative humidity at 800 hPa ($RH_{800}$), (c) rain rate, (d) planetary boundary layer height (PBLH), (e) wind speed at 2 m ($Wind_{2m}$), and (f) wind direction at 2 m ($wind\text{-}dir_{2m}$). Blue and red profiles represent ALEs of DJF and JJA, respectively. Shaded areas show the ALE ranges stemming from the variability of the obtained models from the cross-validation resampling procedure. Markers on the bottom and top x-axes denote the values of $5^{th}$, $25^{th}$, $50^{th}$, $75^{th}$, and $95^{th}$ percentiles for each input variable; note that the first three markers on the x-axes in panel (c) are very close and thus on top of each other.**

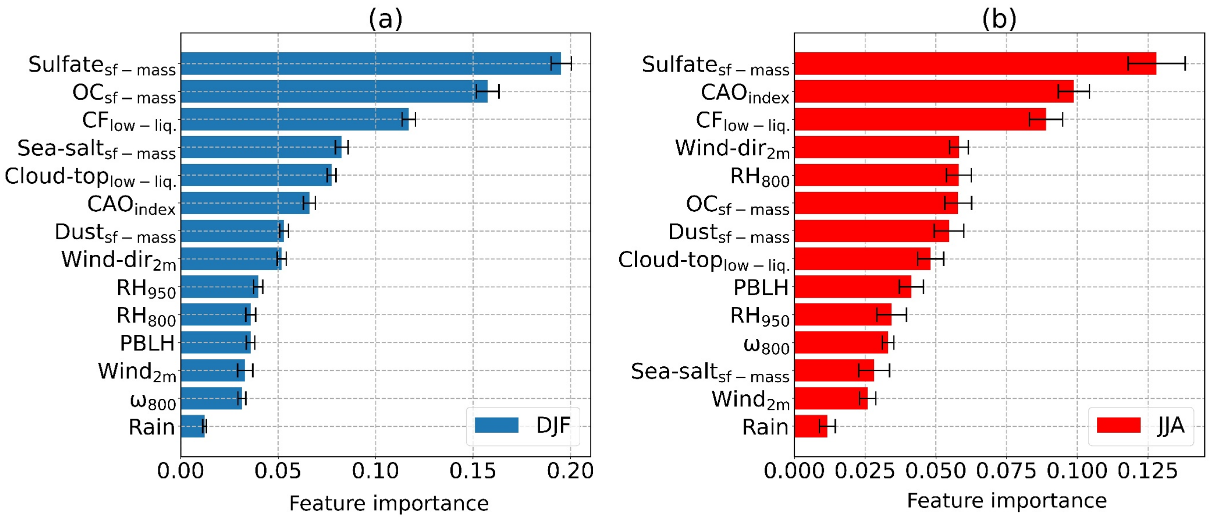


**Figure S22: Average permutation feature importance of input parameters for (a) DJF and (b) JJA based on GBRT models trained in each season on subsets of data including only samples with low-level liquid cloud fraction greater than or equal to 0.7 (i.e., $CF_{low-liq.} \geq 0.7$). Feature importance values were calculated based on using the test set. Error bars exhibit the range of feature importance values stemming from the variability of the obtained models from the cross-validation resampling procedure.**

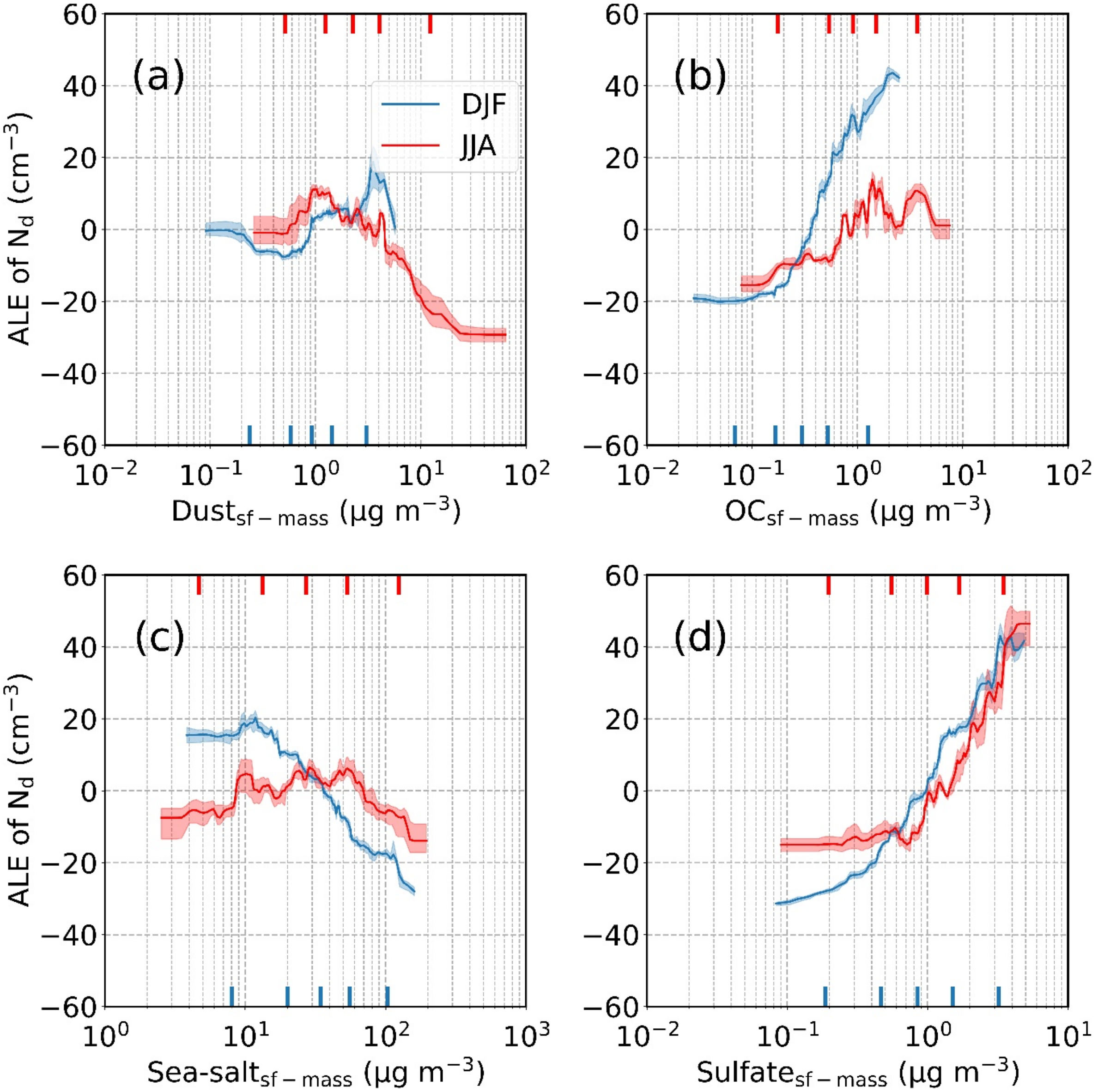


**Figure S23: Average local accumulated effect (ALE) profiles based on GBRT modeling for surface mass concentrations of the following parameters: (a) dust, (b) organic carbon, (c) sea-salt, and (d) sulfate. ALE profiles were based on GBRT modeling on subsets of data including only samples with low-level liquid cloud fraction greater than or equal to 0.7 (i.e., $CF_{low\text{-}liq.} \geq 0.7$). Blue and red profiles represent ALEs of DJF and JJA, respectively. Shaded areas show the ALE ranges stemming from the variability of the obtained models from the cross-validation resampling procedure. Markers on the bottom and top x-axes denote the values of 5$^{th}$, 25$^{th}$, 50$^{th}$, 75$^{th}$, and 95$^{th}$ percentiles for each input variable.**

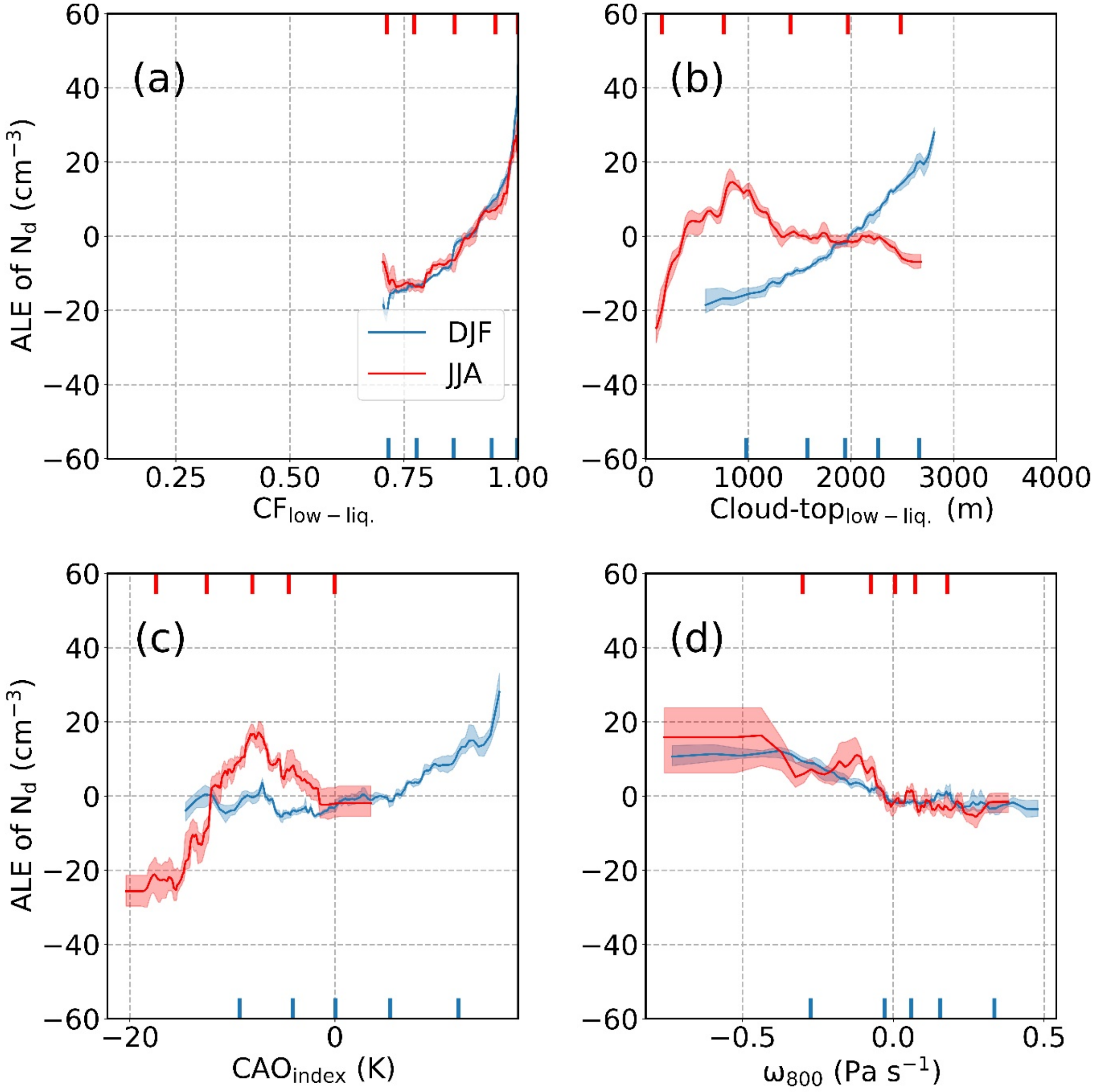


**Figure S24: Same as Figure S23 but for the following input parameters: (a) low-level liquid cloud fraction ($CF_{low\text{-}liq.}$), (b) cloud-top effective height of low-level liquid cloud (cloud-$top_{low\text{-}liq.}$), (c) cold-air outbreak (CAO) index, and (d) vertical pressure velocity at 800 hPa ($\omega_{800}$).**

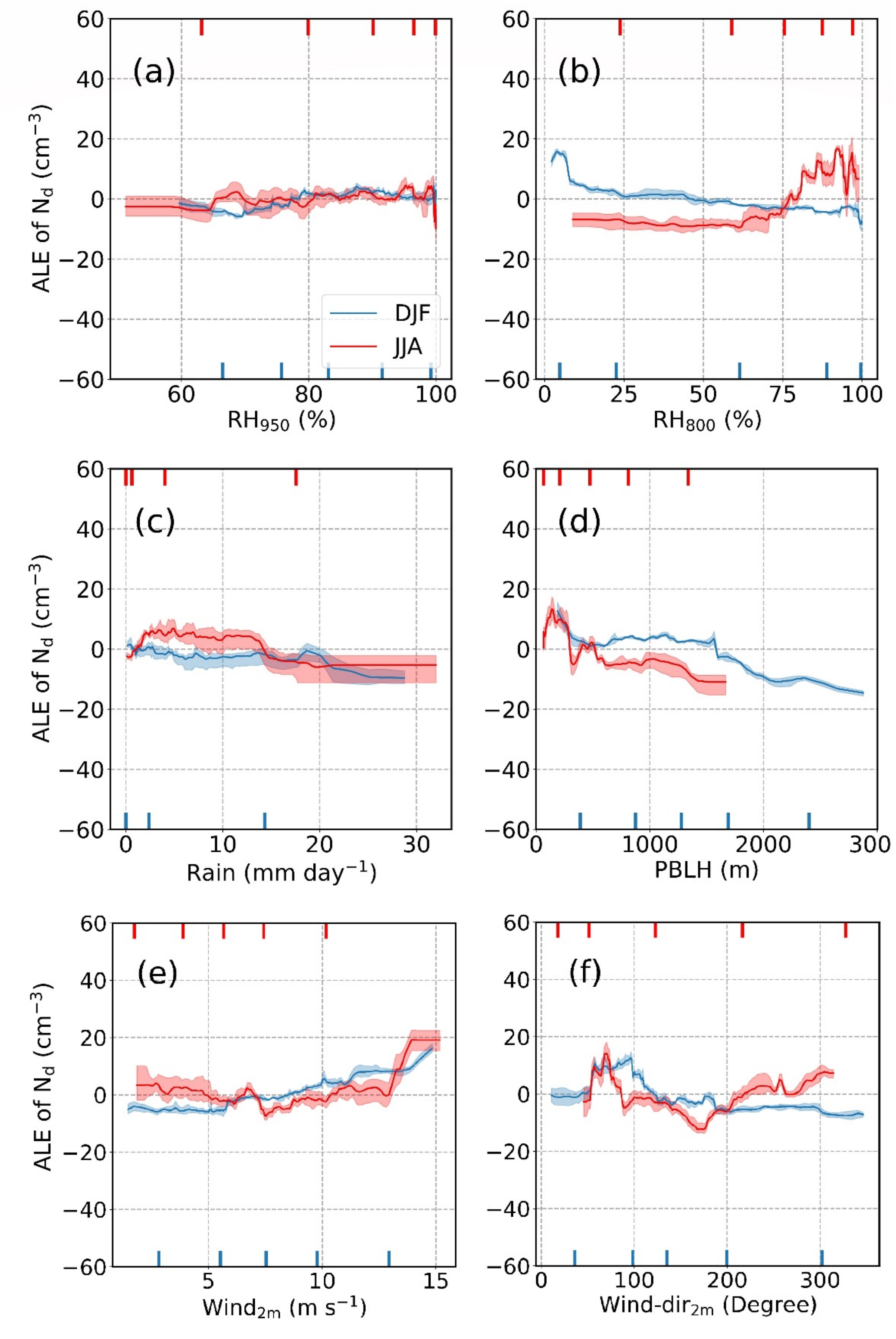


**Figure S25: Same as Figure S23 but for the following input parameters: (a) relative humidity at 950 hPa ($RH_{950}$), (b) relative humidity at 800 hPa ($RH_{800}$), (c) rain rate, (d) planetary boundary layer height (PBLH), (e) wind speed at 2 m ($Wind_{2m}$), and (f) wind direction at 2 m ($wind\text{-}dir_{2m}$).**

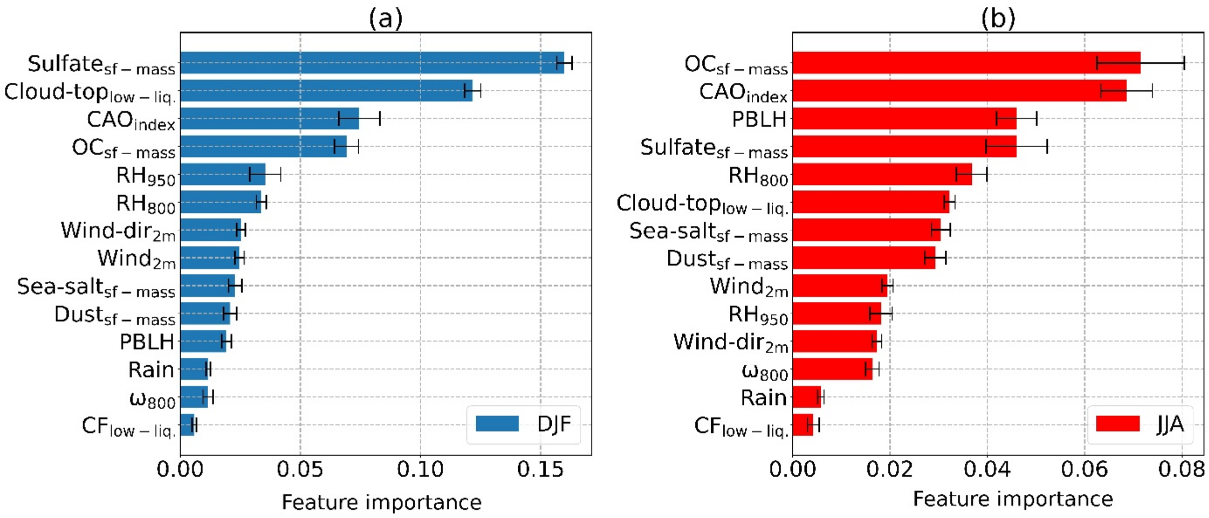


**Figure S26: Average permutation feature importance of input parameters for (a) DJF and (b) JJA based on GBRT models trained in each season on subsets of data including only samples with low-level liquid cloud fraction between 0.2 and 0.4 (i.e., 0.2 ≤ $CF_{low\text{-}liq.}$ ≤ 0.4). Feature importance values were calculated based on using the test set. Error bars exhibit the range of feature importance values stemming from the variability of the obtained models from the cross-validation resampling procedure.**

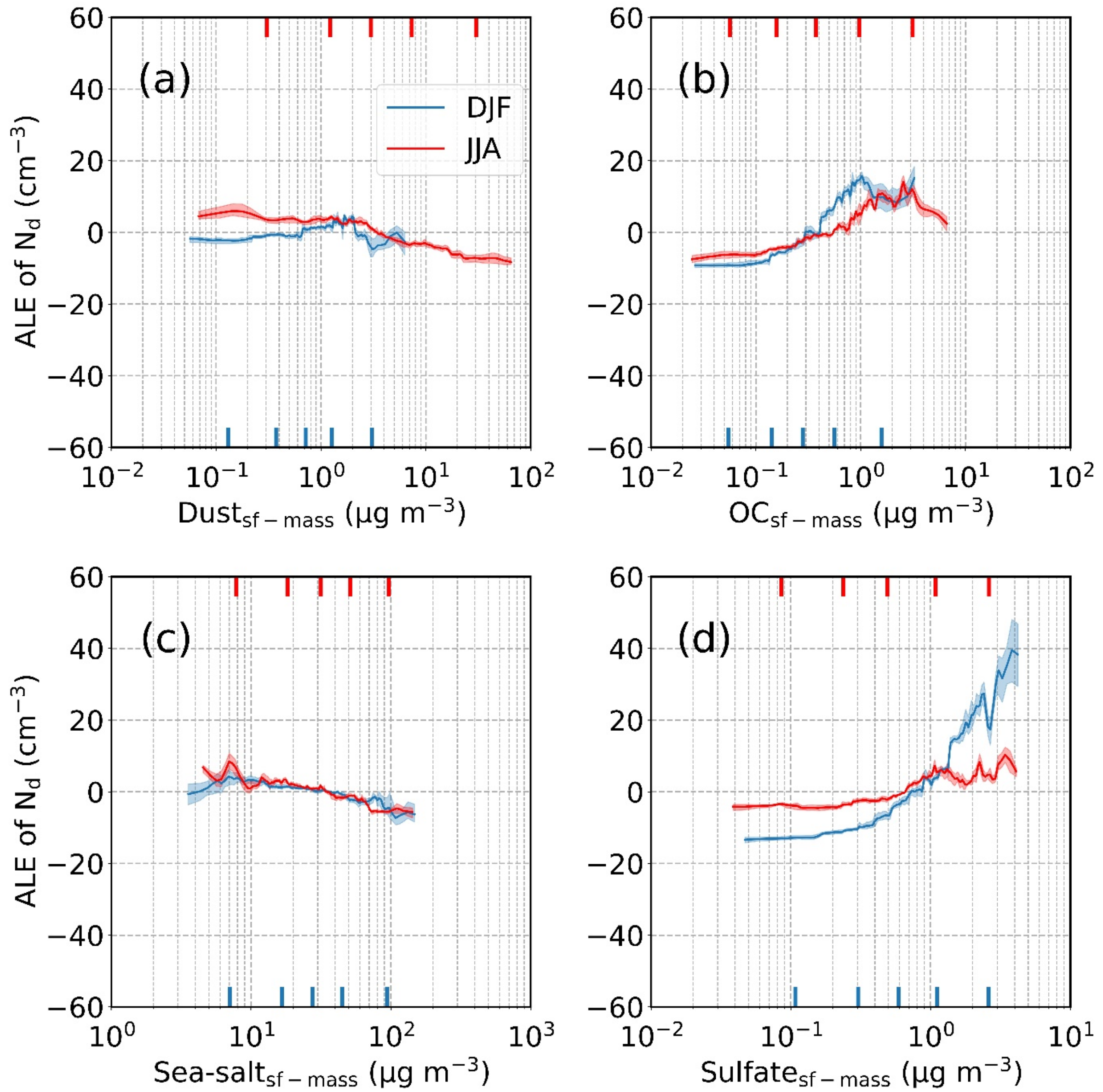


**Figure S27: Average local accumulated effect (ALE) profiles based on GBRT modeling for surface mass concentrations of the following parameters: (a) dust, (b) organic carbon, (c) sea-salt, and (d) sulfate. ALE profiles were based on GBRT modeling on subsets of data including only samples with low-level liquid cloud fraction between 0.2 and 0.4 (i.e., $0.2 \leq CF_{low\text{-}liq.} \leq 0.4$). Blue and red profiles represent ALEs of DJF and JJA, respectively. Shaded areas show the ALE ranges stemming from the variability of the obtained models from the cross-validation resampling procedure. Markers on the bottom and top x-axes denote the values of 5$^{th}$, 25$^{th}$, 50$^{th}$, 75$^{th}$, and 95$^{th}$ percentiles for each input variable.**

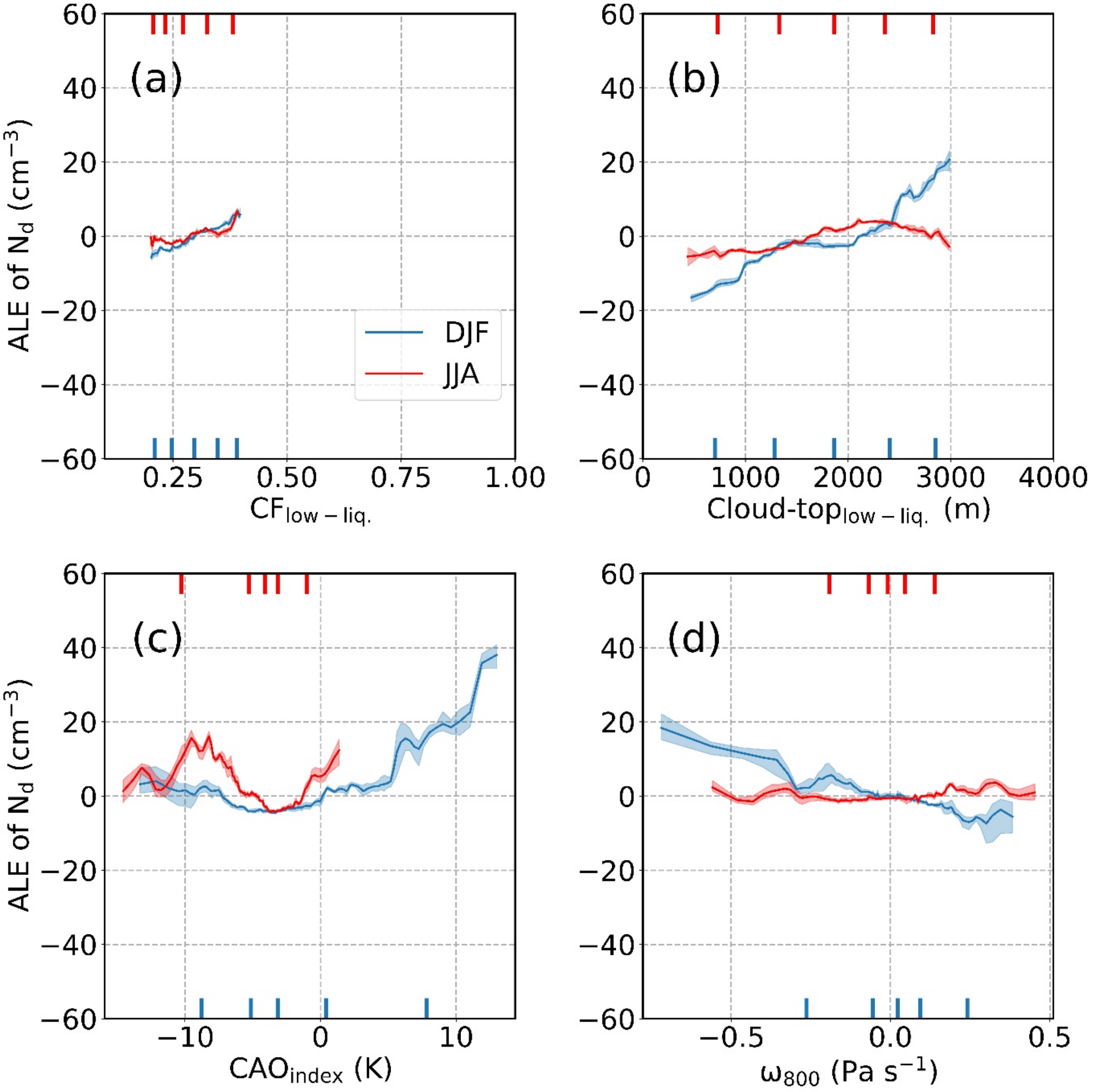


**Figure S28: Same as Figure S27 but for the following input parameters: (a) low-level liquid cloud fraction ($CF_{low\text{-}liq.}$), (b) cloud-top effective height of low-level liquid cloud ($cloud\text{-}top_{low\text{-}liq.}$), (c) cold-air outbreak (CAO) index, and (d) vertical pressure velocity at 800 hPa ($\omega_{800}$).**

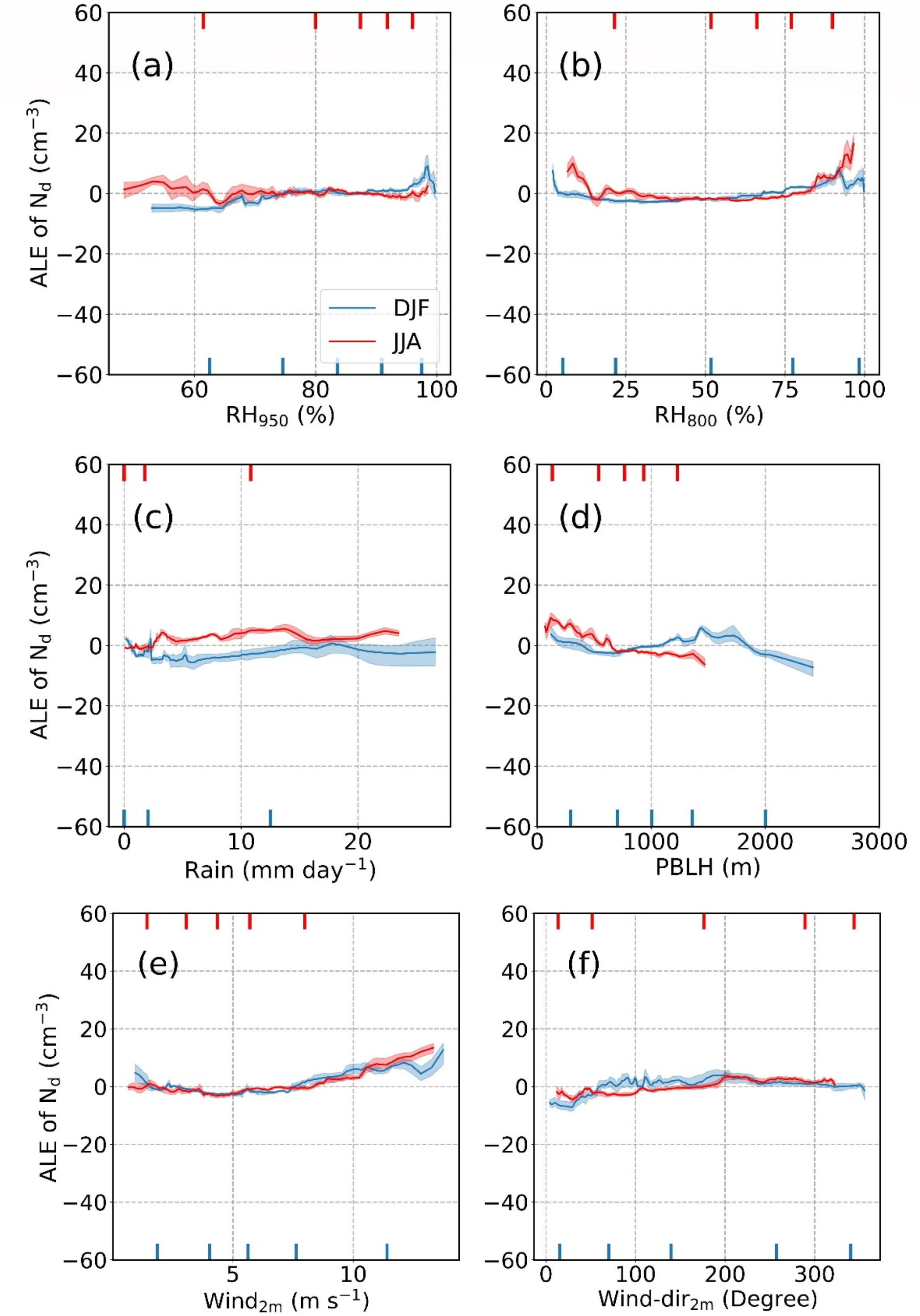


**Figure S29: Same as Figure S27 but for the following input parameters: (a) relative humidity at 950 hPa ($RH_{950}$), (b) relative humidity at 800 hPa ($RH_{800}$), (c) rain rate, (d) planetary boundary layer height (PBLH), (e) wind speed at 2 m ($Wind_{2m}$), and (f) wind direction at 2 m (wind-$dir_{2m}$).**
